# Cancer-associated fibroblasts secrete CSF3 to promote TNBC progression via enhancing PGM2L1-dependent glycolysis reprogramming

**DOI:** 10.1038/s41419-025-07580-6

**Published:** 2025-04-04

**Authors:** Wenqi Qin, Bing Chen, Xin Li, Wenjing Zhao, Lijuan Wang, Ning Zhang, Xiaolong Wang, Dan Luo, Yiran Liang, Yaming Li, Xi Chen, Tong Chen, Qifeng Yang

**Affiliations:** 1 Department of Breast Surgery, General Surgery, Qilu Hospital of Shandong University, Jinan, Shandong PR China; 2 Biological Resource Center, Qilu Hospital of Shandong University, Jinan, Shandong PR China; 3 Research Institute of Breast Cancer, Shandong University, Jinan, Shandong PR China

**Keywords:** Breast cancer, Mechanisms of disease

## Abstract

Triple-negative breast cancer (TNBC) is characterized by a pronounced hypoxic tumor microenvironment, with cancer-associated fibroblasts (CAFs) serving as the predominant cellular component and playing crucial roles in regulating tumor progression. However, the mechanism by which CAFs affect the biological behavior of tumor cells in hypoxic environment remain elusive. This study employed a bead-based multiplex immunoassay to analyze a panel of cytokines/chemokines and identified colony stimulating factor 3 (CSF3) as a significantly elevated component in the secretome of hypoxic CAFs. We found that CSF3 promoted the invasive behavior of TNBC cells by activating the downstream signaling pathway of its receptor, CSF3R. RNA sequencing analysis further revealed that phosphoglucomutase 2-like 1 (PGM2L1) is a downstream target of the CSF3/CSF3R signaling, enhancing the glycolysis pathway and providing energy to support the malignant phenotype of breast cancer. In vivo, we further confirmed that CSF3 promotes TNBC progression by targeting PGM2L1. These findings suggest that targeting CSF3/CSF3R may represent a potential therapeutic approach for TNBC.

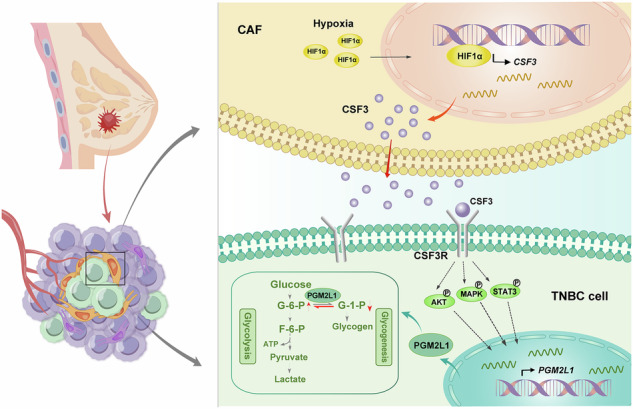

## Introduction

The tumor microenvironment (TME), composed of tumor cells, stromal and immune cells, and various secreted factors, is identified as a critical driver of tumor progression and metastasis [1]. Among the stromal components, cancer-associated fibroblasts (CAFs) are the most abundant and play a key role in creating an abnormal TME that supports cancer progression [2]. Evidences show that CAFs participate in crosstalk with tumor cells by secreting cytokines and chemokines, influencing both therapeutic response and cancer prognosis. This highlights the predictive and prognostic value of CAFs and their secreted factors [3, 4].

Hypoxia is a hallmark of most solid tumors and a common characteristic of TME, associated with a more aggressive tumor cell phenotype. This is primarily due to the fact that angiogenesis frequently lags behind tumor proliferation and abnormal neovascularization [5, 6]. In breast cancer (BC), hypoxia is more pronounced in triple-negative breast cancer (TNBC) compared to other subtypes [7, 8], complicating treatment and suggesting the need for further research and combination therapies [9]. The CAFs are highly sensitive to hypoxia and participate in crosstalk with cancer cells, promoting malignancy [10, 11]. However, the roles of hypoxic CAFs and their secreted factors in driving TNBC progression, along with the underlying mechanisms, remain largely unexplored.

In our current study, through in vitro and in vivo experiments, we discovered that colony stimulating factor 3 (CSF3) secreted by hypoxic CAFs promoted invasive behavior of TNBC cells by enhancing phosphoglucomutase 2-like 1 (PGM2L1)-dependent glycolysis reprogramming. These findings shed light on the role of hypoxic CSF3 in TNBC progression and its underlying mechanism, offering potential therapeutic targets for TNBC treatment.

## Results

### CAFs promote the proliferation, migration, and invasion of TNBC cells under hypoxic conditions

**Fig. 1:**
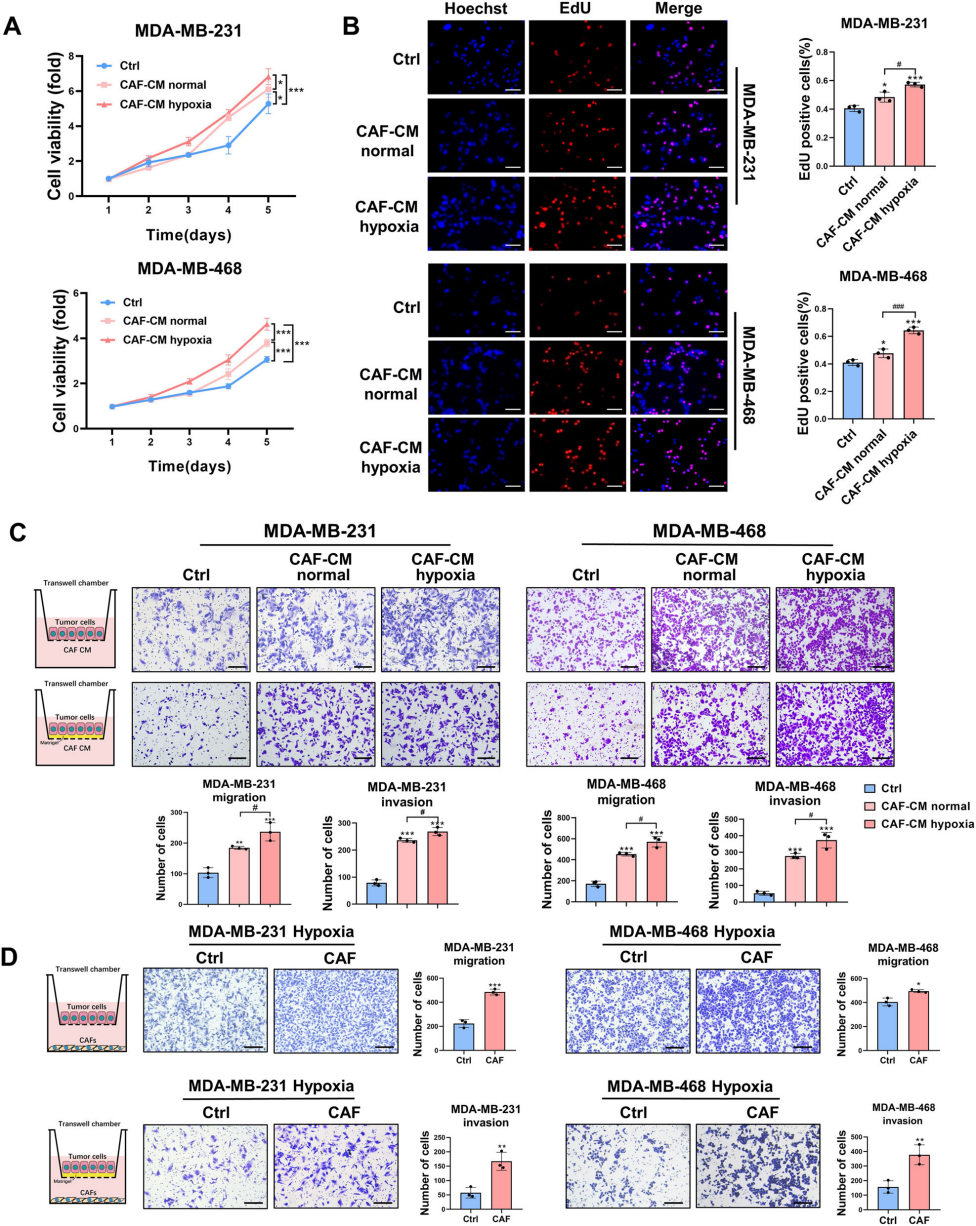
CAFs promote the proliferation, migration, and invasion of TNBC cells under hypoxic conditions. The proliferation ability of TNBC cells treated with CAF-CM was detected by MTT assay (**A**, n = 4) and EdU assay (**B**, n = 3). Scale bars, 100 μm. **C** Transwell assay was performed to evaluate the effect of normoxic or hypoxic CAF-CM on the migration and invasion abilities of TNBC cells (n = 3). Scale bars, 200 μm. **D** Transwell assay was performed to evaluate the effect of CAFs on the migration and invasion abilities of TNBC cells under hypoxia (n = 3). Scale bars, 200 μm. The data are presented as the mean ± SD. *^,#^*P* < 0.05, **P < 0.01, ***^,###^*P* < 0.001.

CAFs were isolated from breast carcinoma tissues acquired from TNBC patients. The obtained CAFs showed typical fibroblast morphology (spindle-shaped and fascicular). To validate the characteristics of these CAFs, normal fibroblasts (NFs) were isolated from adjacent normal breast tissue. CAFs showed a significant increase in the expression of CAF markers, including α-smooth muscle actin (α-SMA), fibroblast activation protein (FAP), and fibroblast-specific protein 1 (FSP1), compared to NFs (Supplementary Fig. 1A, B). TNBC is characterized by a pronounced hypoxic environment. To assess the effect of CAFs on the malignant behaviors of cancer cells under hypoxic conditions, a conditioned medium (CM) was collected from CAFs cultured in normoxic and hypoxic conditions for 48 h, respectively. MTT and EdU assays revealed that the addition of CAF-CM can significantly increase the proliferation level of TNBC cells, and hypoxia can further enhance this trend. Understanding how they affect cell viability would provide valuable insight (Fig. 1A, B).

To simulate the TME involving TNBC cells and CAFs, two non-contact co-culture models using transwell chambers were employed. In the first model, TNBC cells were seeded in the upper chamber and cultured with either normoxic or hypoxic CAF-CM. The results demonstrated that TNBC cells treated with normoxic CAF-CM showed enhanced migration and invasion abilities than cells treated with ordinary medium. One of the main reasons is that the CAF-CM contains a large number of cytokines. The hypoxic CAF-CM further increased the migration and invasion of cells (Fig. 1C). In the second model, CAFs were plated in the lower chamber and TNBC cells were placed in the upper chamber, and cultured them under hypoxic conditions. The results indicated that TNBC cells co-cultured with CAFs under hypoxic conditions displayed a stronger capacity for migration and invasion (Fig. 1D). These results collectively suggested that CAFs promoted the proliferation, migration, and invasion of TNBC cells under a hypoxic conditions.

### Hypoxia induces the secretion of CSF3 from CAFs

**Fig. 2:**
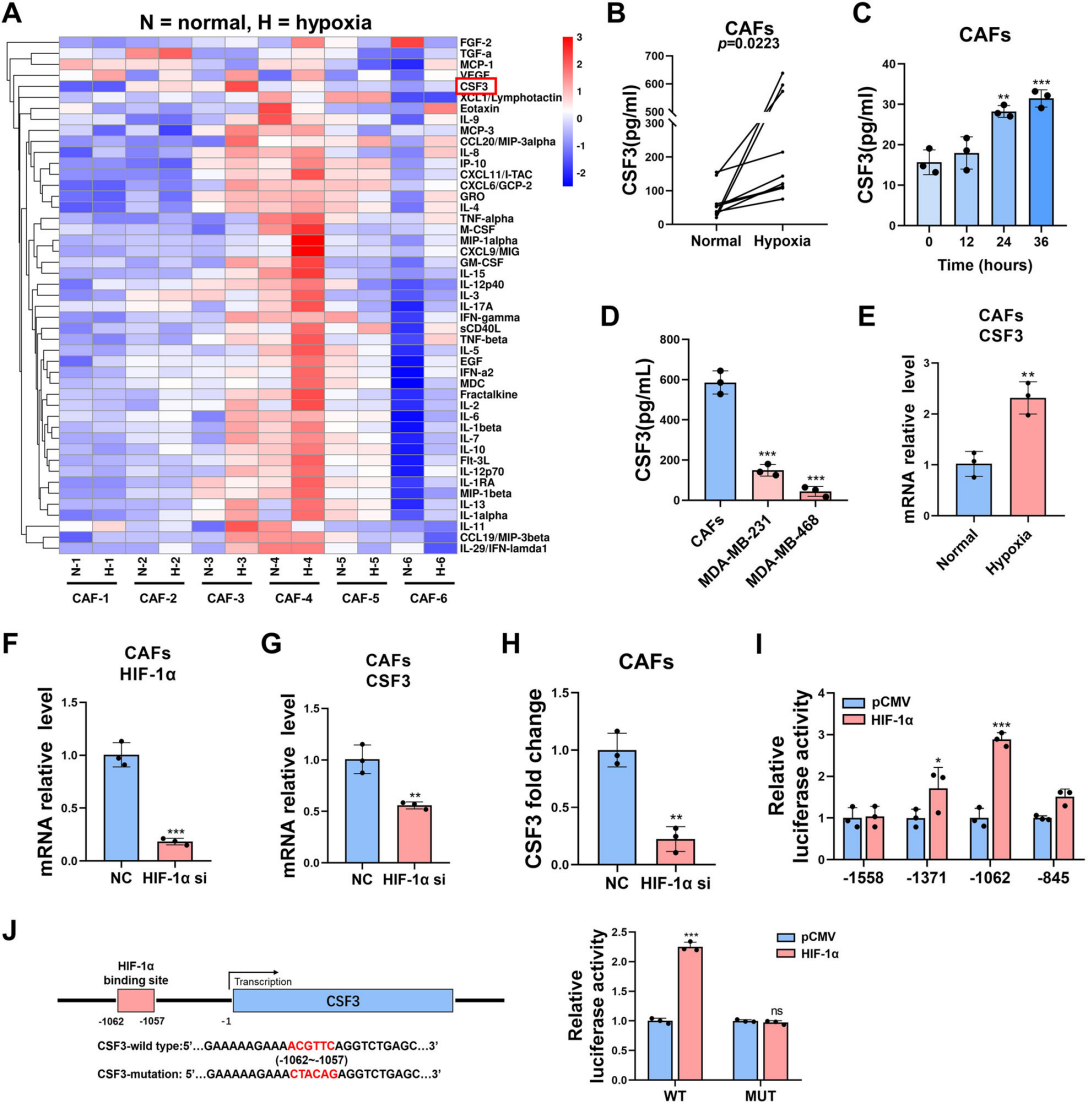
Hypoxia induces the secretion of CSF3 from CAFs. **A** The bead-based multiplex immunoassay assay to test cytokines secreted by CAFs under normoxic and hypoxic conditions (n = 6). **B** ELISA assay to quantify CSF3 secreted from paired CAFs under normoxic and hypoxic conditions (n = 10). **C** The secretion level of CSF3 increases with the prolonged hypoxic exposure (n = 3). **D** The comparation of CSF3 level from CAFs and TNBC cells (n = 3). **E** The expression of CSF3 under normoxic and hypoxic conditions was assessed by qRT-PCR (n = 3). The HIF-1α silencing efficiency (**F**) and CSF3 expression level (**G**) were verified using qRT-PCR (n = 3). **H** ELISA assay to test CSF3 level after interfering HIF-1α (n = 3). **I** The luciferase activity of different targeted plasmids containing potential binding region (n = 3). **J** Dual-luciferase reporter assay to verify CSF3’ binding region for HIF-1α (n = 3). The data are presented as the mean ± SD. ns, no significance; *P < 0.05, **P < 0.01, ***P < 0.001.

A primary mechanism by which CAFs promote tumor cells progression is through the secretion of cytokines and chemokines. To identify the factors secreted by CAFs under hypoxic conditions, six pairs of CAFs were cultured under either normoxic or hypoxic conditions for 48 h, and CM was collected. Then a panel of cytokines/chemokines within the CM was measured using a bead-based multiplex immunoassay (Merck Millipore), which enables the simultaneous analysis of 47 different cytokines. Differential secretome profiles between normoxic and hypoxic CAFs were observed (Fig. 2A). Four cytokines were found to be increased under hypoxic conditions: CCL20, GM-CSF, CSF3, and VEGF. The secretion levels of CCL20 and GM-CSF were relatively lower than that of CSF3 and VEGF. Literatures have reported that VEGF promotes angiogenesis to adapt to hypoxic environments [12, 13]. Therefore, the focus shifted to CSF3. To confirm that CAFs secrete higher levels of CSF3 under hypoxia, their levels were analyzed in additional paired CAFs samples cultured under normoxic and hypoxic conditions (Fig. 2B). ELISA results demonstrated that CSF3 secreted by CAFs increased with prolonged hypoxic exposure (Fig. 2C). Furthermore, ELISA assays showed that CSF3 levels were higher in CAFs compared to TNBC cells, indicating that CAFs were one of the main sources of CSF3 under hypoxic conditions (Fig. 2D).

In order to investigate the mechanism behind increased CSF3 secretion from CAFs under hypoxic conditions, the mRNA levels of CSF3 were assessed. qRT-PCR analysis revealed that CSF3 expression was significantly higher in CAFs under hypoxic conditions compared to normoxic conditions (Fig. 2E). Hypoxia-inducible factor-1α (HIF-1α) is a pivotal regulatory factor in adapting to hypoxic conditions, and numerous genes have been identified as direct transcriptional targets of HIF-1α in CAFs or cancer cells [14, 15]. Consequently, we sought to determine if HIF-1α regulates CSF3 transcription. To verity this hypothesis, HIF-1α was silenced in CAFs using siRNA. The qRT-PCR analysis confirmed the effective silencing of HIF-1α expression in CAFs (Fig. 2F) and revealed a significant decrease in CSF3 mRNA levels (Fig. 2G). ELISA results further demonstrated that silencing HIF-1α in CAFs led to a significant reduction in CSF3 transcription (Fig. 2H). Furthermore, four potential binding sites of HIF-1α within the 5’ UTR of CSF3 were predicted using JASPAR. Luciferase reporter assays were performed and verified that the CSF3 5’ UTR region, spanning −1062 to −1057 bp upstream of the transcription start site, may serves as the binding site for HIF-1α involved in transcriptional regulation (Fig. 2I). To further investigate, dual-luciferase reporter vectors with either the wild type (WT) or mutated (MUT) 5’ UTR region were constructed for luciferase reporter assay (Fig. 2J). A significant increase in luciferase activity was observed in 293T cells co-transfected with HIF-1α and the WT vector, but not with the MUT vector (Fig. 2J). This indicated that HIF-1α may regulate CSF3 transcription by binding to the -1062 to -1057 bp region of its 5’ UTR. Therefore, the increased CSF3 secretion from CAFs under hypoxic conditions is likely attributable to HIF-1α-mediated transcriptional regulation of CSF3.

### CSF3/CSF3R promotes the proliferation, migration, and invasion of TNBC cells

**Fig. 3:**
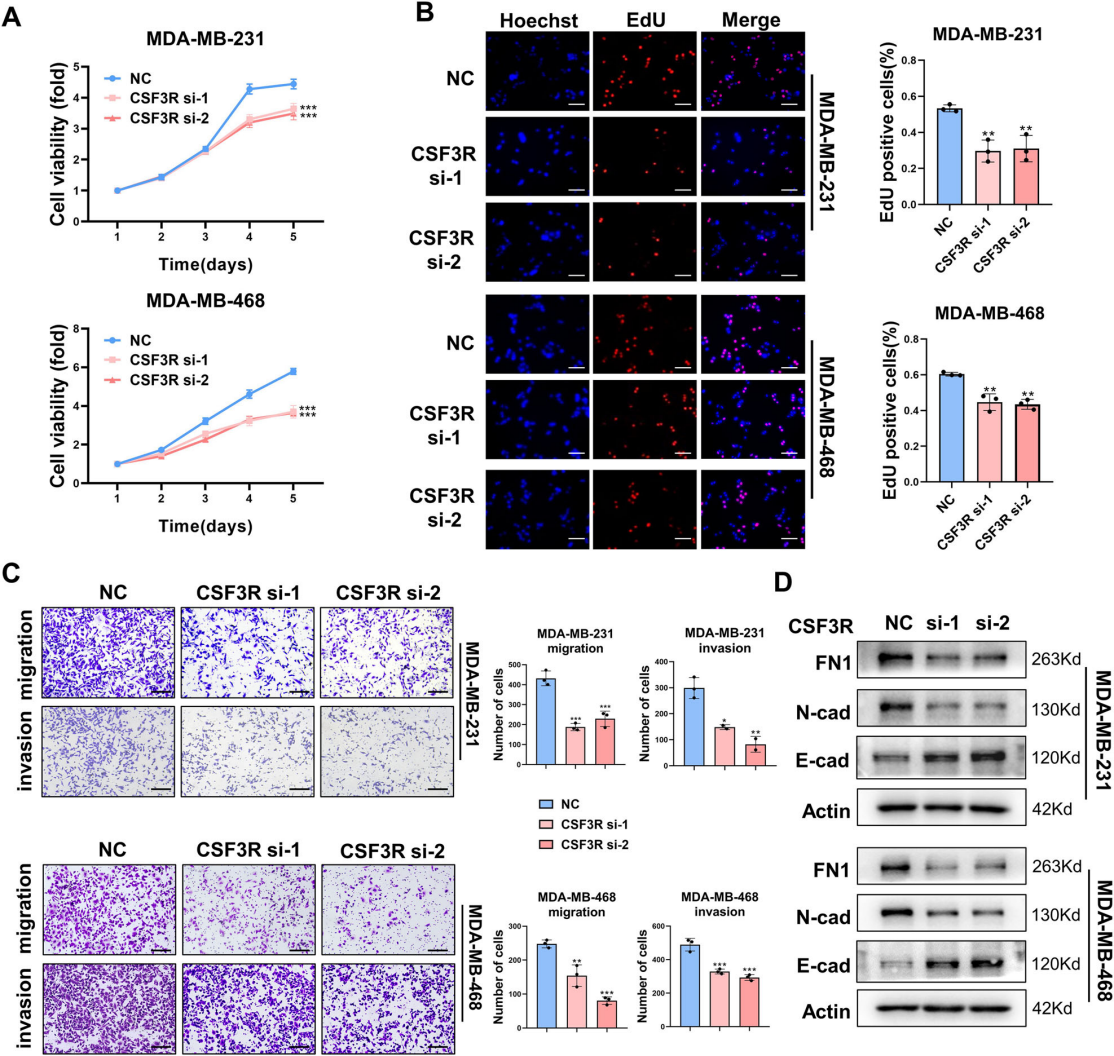
CSF3/CSF3R promotes the proliferation, migration, and invasion of TNBC cells. The proliferation ability of TNBC cells silencing CSF3R was determined by MTT assay(**A**, n = 4) and EdU assay (**B**, n = 3). Scale bars, 100 μm. **C** Transwell assay was implemented to assessed the migration and invasion abilities of TNBC cells silencing CSF3R (n = 3). Scale bars, 200 μm. **D** The expression of EMT-related markers was examined via western blot. The data are presented as the mean ± SD. *P < 0.05, **P < 0.01, ***P < 0.001.

The diverse biological effects of CSF3 are known to be mediated through its specific cell surface receptor, CSF3R [16]. MDA-MB-231 and MDA-MB-468 cells were cultured under hypoxic conditions for different durations, and the expression of CSF3R was assessed. The results showed that both mRNA and protein levels of CSF3R increased in TNBC cells with prolonged hypoxia (Supplementary Fig. 2A, B). To investigate whether CSF3 promotes TNBC proliferation and metastasis through CSF3R under hypoxic conditions, siRNA was used to knock down CSF3R expression in TNBC cells (Supplementary Fig. 2C, D). Then, MTT and EdU assays revealed that TNBC cells transfected with CSF3R siRNA showed reduced growth compared to the control group when treated with CAF-CM under hypoxia (Figs. 3A, B). Furthermore, the migration and invasion abilities of TNBC cells were significantly inhibited following CSF3R silencing (Fig. 3C). Given the established role of epithelial-mesenchymal transition (EMT) in tumor cell migration, we investigated the expression of EMT markers in TNBC cells. The results demonstrated that CSF3R knockdown decreased the expression of mesenchymal markers, Fibronectin (FN1) and N-cadherin (N-cad), while increasing the expression of epithelial marker, E-cadherin (E-cad) (Fig. 3D).

Ligand-induced dimerization of CSF3R rapidly activates downstream signal transduction pathways, primarily involving the Ras/MAPK (mitogen-activated protein kinase), JAK/STAT (Janus kinase/signal transducer and activator of transcription), and Lyn-PI3K/Akt (phosphatidylinositol 3-kinase/Akt) pathways [16, 17]. We confirmed the activation of these signaling pathways in hypoxic TNBC cells treated with CAF-CM (Supplementary Fig. 2E). These findings indicated that CSF3 derived from CAFs promoted tumor progression through the CSF3/CSF3R signaling axis in TNBC cells.

### PGM2L1 is a downstream regulatory target of CSF3/CSF3R signaling

**Fig. 4:**
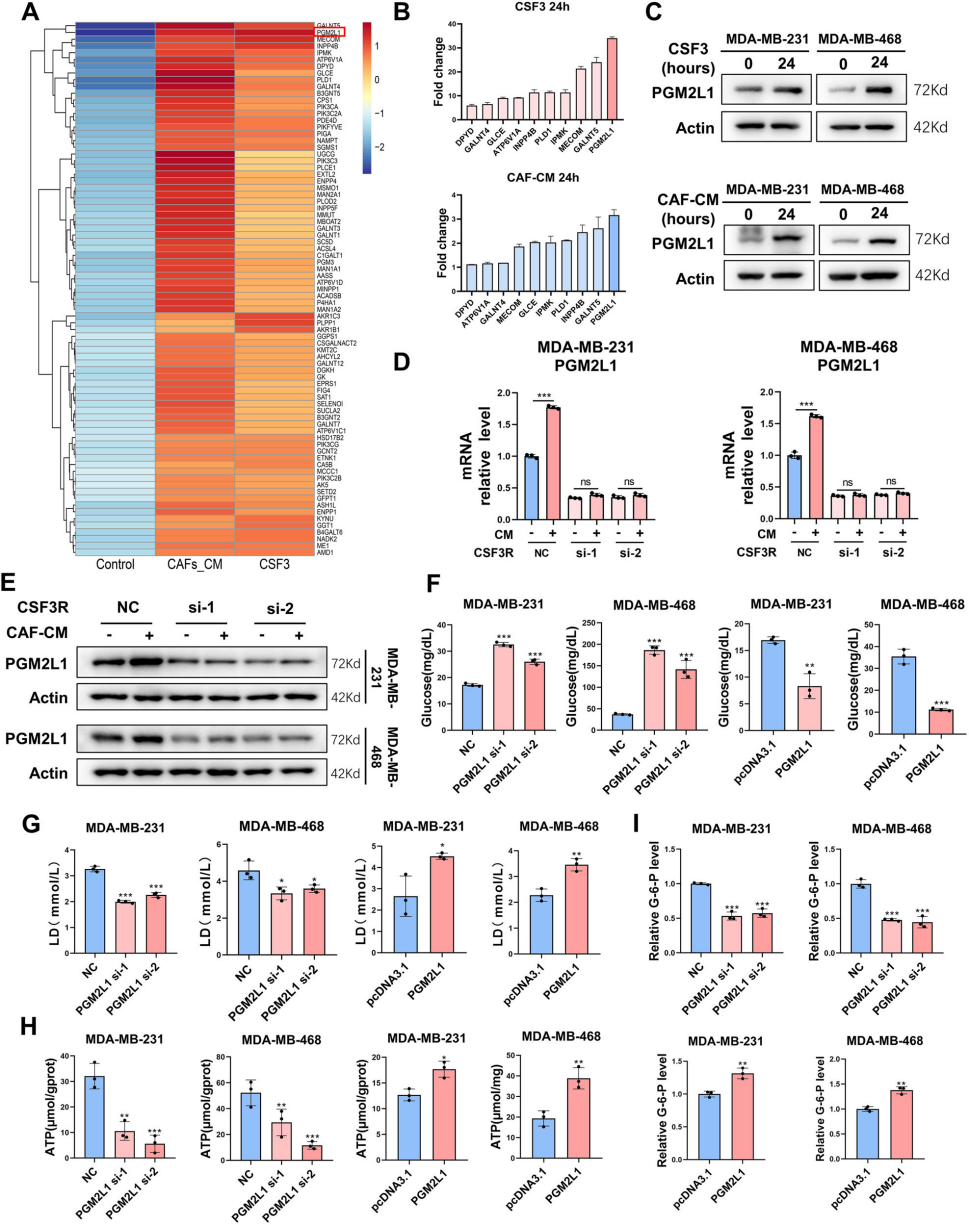
PGM2L1 is a downstream regulatory target of CSF3/CSF3R signaling. **A** Heat map of top 80 metabolic pathways-related DEGs in both CSF3 and CAF-CM groups. **B** The expression level of top differential genes by qRT-PCR (n = 3). **C** The protein level of PGM2L1 was upregulated by CSF3 and CAF-CM. The expression level of PGM2L1 was decreased by silencing CSF3R in mRNA level (**D**, n = 3) and protein level (**E**). Colorimetry assays to measure glycolysis of MDA-MB-231 and MDA-MB-468 cells under hypoxia, including glucose (**F**), lactic acid (**G**), ATP (**H**), and G-6-P (**I**) (n = 3). The data are presented as the mean ± SD. ns, no significance; *P < 0.05, **P < 0.01, ***P <0.001.

To elucidate the mechanism underlying CSF3/CSF3R function in TNBC cells, RNA-seq analysis was performed to identify potential genes contributing to the oncogenic effects of CSF3. MDA-MB-231 cells were treated with hypoxic CAF-CM or recombinant human CSF3 (rhCSF3, 50 µg/mL). Kyoto Encyclopedia of Genes and Genomes (KEGG) enrichment analysis was performed on the differentially expressed genes (DEGs) identified by RNA-seq. As depicted in Supplementary Fig. 3A, B, the DEGs were significantly enriched in metabolic pathways, with metabolism-related genes in TNBC cells markedly upregulated under the influence of CSF3 and CAF-CM. Further analysis of the metabolic pathways-related genes revealed that 108 genes were elevated in MDA-MB-231 cells treated with both CSF3 and CAF-CM (Supplementary Fig. 3C). The top 80 DEGs related to metabolic pathways were presented in the heat map (Fig. 4A). The most significantly altered genes were then validated using qRT-PCR, revealing that the expression of PGM2L1 was most significantly increased in TNBC cells treated with CSF3 and CAF-CM (Fig. 4B). The protein expression of PGM2L1 mirrored its mRNA levels (Fig. 4C). In contrast, silencing CSF3R in TNBC cells eliminated the CAF-CM-induced upregulation of PGM2L1 mRNA and protein expression (Fig. 4D, E). Then, inhibitors targeting the ERK, STAT, and AKT signaling pathways were used separately. The results indicated that inhibiting any single pathway alone did not noticeably decrease PGM2L1 expression, but the combined use of all three inhibitors significantly reduced the CSF3-induced upregulation of PGM2L1 (Supplementary Fig. 3E). Therefore, we hypothesized that PGM2L1 may be co-regulated by the three downstream signaling pathways of CSF3R.

Subsequently, we investigated the impact of CSF3 and CAF-CM on the expression of key glycolysis-related genes in TNBC cells, including PFKL, ENO1, PKM1F, HK2, PKM2, and PDK1. As shown in Supplementary Fig 3F, the expression of these six glycolysis-related genes was not significantly altered by CSF3 and CAF-CM, suggesting that CSF3 secreted by CAFs particularly influenced the expression of PGM2L1 without affecting the overall expression of glycolysis-related genes. Current studies have demonstrated that PGM2L1 is associated with poor prognosis in patients with gastric cancer and prostate cancer [18, 19]. However, the role and potential mechanisms of PGM2L1 in TNBC cells remain unclear and will be the primary focus of future research.

### CAFs promote TNBC progression via PGM2L1-mediated reprogramming of glycolysis

To explore the clinical characteristics of PGM2L1 in BC, qRT-PCR was performed on samples from a cohort of patients at Qilu Hospital, revealing that PGM2L1 expression was upregulated in BC tissues compared to normal tissues (Supplementary Fig. 4A). This finding was corroborated by the analysis of high-throughput RNA-seq data from TCGA database (Supplementary Fig. 4B). Higher expression of PGM2L1 was also observed in both TNBC (Supplementary Fig. 4C) and metastatic TNBC tumors (Supplementary Fig. 4D). Furthermore, immunohistochemical staining confirmed elevated PGM2L1 expression in TNBC tissues compared to normal tissues (Supplementary Fig. 4E). Kaplan-Meier survival analysis indicated that higher PGM2L1 expression was associated with poorer overall survival in all BC types specifically in the TNBC subtype (Supplementary Fig. 4F, G). These findings suggested that PGM2L1 may play an oncogenic role in BC progression and is particularly linked to the TNBC subtype.

**Fig. 5:**
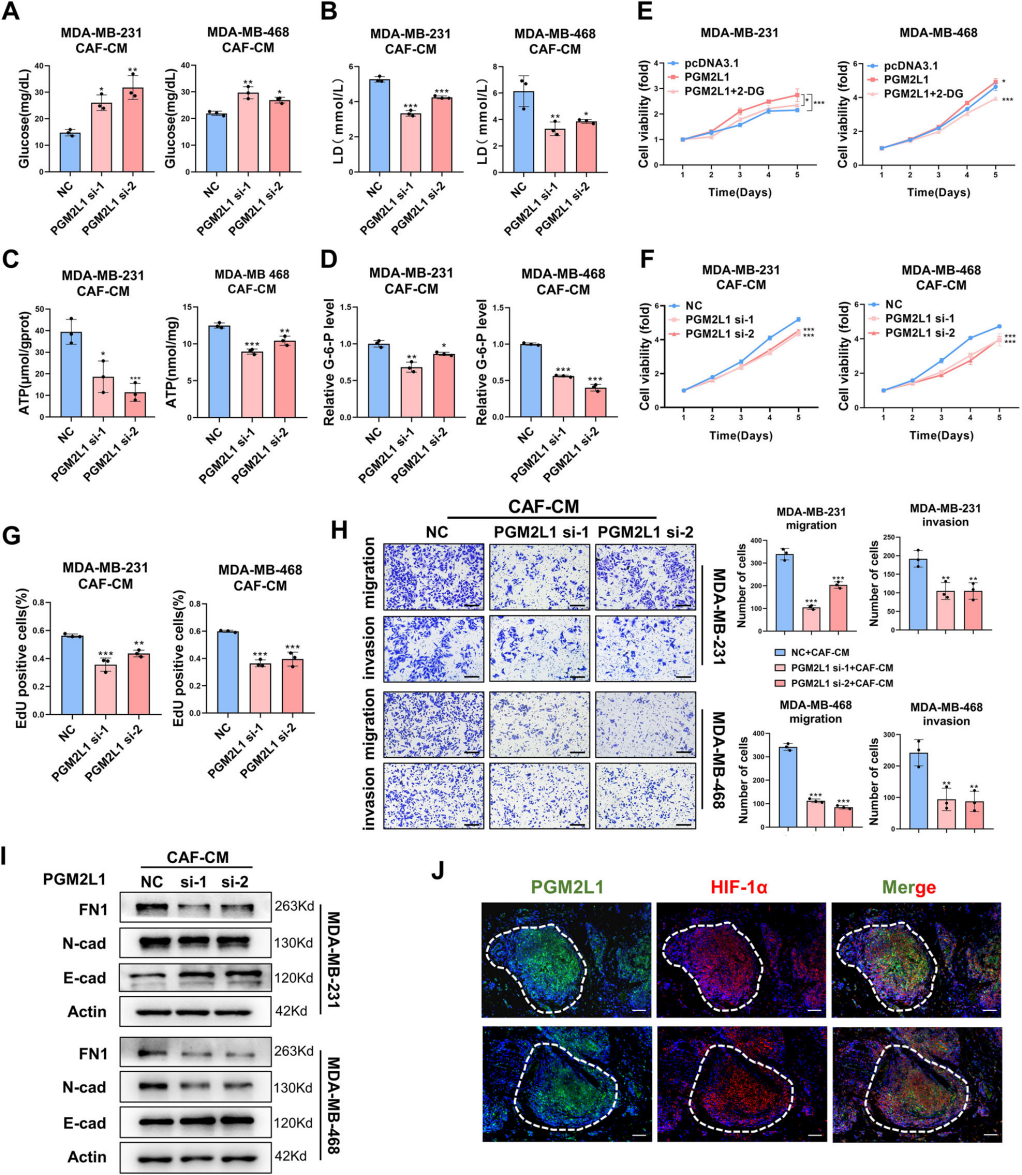
PGM2L1 promotes the glycolysis and progression of TNBC cells under hypoxia. Colorimetry assays to measure glycolysis level of MDA-MB-231 and MDA-MB-468 cells treated with hypoxic CAF-CM, including glucose (**A**), lactic acid (**B**), ATP (**C**), and G-6-P(**D**) (n = 3). **E** MTT assay to assess cellular proliferation with or without 2-DG (n = 4). MTT (**F**, n = 4) and EdU (**G**, n = 3) assays to evaluate the proliferative ability of TNBC cells treated with hypoxic CAF-CM following PGM2L1 silencing. **H** Transwell assay to assess the migration and invasion abilities of TNBC cells treated with hypoxic CAF-CM following PGM2L1 silencing (n = 3). Scale bars, 200 μm. **I** The EMT-makers were examined by western blot. **J** Fluorescent immunohistochemical staining of the expression of PGM2L1 and HIF-1α in TNBC tissues. Scale bars, 100 μm. The data are presented as the mean ± SD. *P < 0.05, **P < 0.01, ***P < 0.001.

To further investigate the biological impact of PGM2L1 on TNBC cells under hypoxic conditions, PGM2L1 was either overexpressed using the pcDNA3.1-PGM2L1 plasmid or silenced with siRNA (Supplementary Fig. 5A, B, Supplementary Fig. 6A, B). Gain- and loss-of-function experiments were then conducted. The results of MTT assay and EdU assay revealed that overexpressing PGM2L1 enhanced the proliferative ability of TNBC cells (Supplementary Fig. 5C, D). Similarly, silencing PGM2L1 impaired their proliferative ability, as demonstrated in Supplementary Fig. 6C, D. Transwell assay results demonstrated that PGM2L1 overexpression was essential for maintaining the migration and invasion capabilities of TNBC cells, while PGM2L1 silencing had the opposite effect (Supplementary Figs. 5E and 6E). Moreover, PGM2L1 was found to regulate the EMT process in TNBC cells (Supplementary Figs. 5F and 6F). In addition, to confirm the relationship between PGM2L1 and hypoxia, we conducted fluorescent immunohistochemical staining of TNBC tissue slices. The fluorescence revealed the co-expression of PGM2L1 and HIF-1α in the central region of the tumor, which was considered relatively hypoxic regions (Fig. 5G). These findings suggested that PGM2L1 played a crucial role in the progression of TNBC cells under hypoxic conditions. The fluorescence also showed obvious consistency between the position of CSF3R and HIF-1α expression, further demonstrating the correlation between CSF3R expression and hypoxic conditions (Supplementary Fig. 5H).

PGM2L1 is a member of the PGM (phosphoglucomutase) family, which primarily functions to facilitate the interconversion between glucose-1-phosphate (G-1-P) and glucose-6-phosphate (G-6-P). In glucose metabolism, G-1-P is involved in glycogen synthesis, while G-6-P is associated with glycolysis. Consequently, members of the PGM family play critical roles in regulating the metabolic balance between glycogen synthesis and glycolysis (Supplementary Fig. 3D). In hypoxic microenvironments, cancer cells tend to favor glycolysis, as increased glycolysis provides more intermediate products and precursor molecules for biosynthesis, as well as energy to support tumor progression. Hence, it is hypothesized that PGM2L1 promotes glycolysis rather than glycogen synthesis under hypoxic conditions.

Glucose undergoes the glycolysis process to be transformed into pyruvate, which is further converted into lactic acid in the hypoxic microenvironment, yielding ATP in the process (Supplementary Fig. 3D). Therefore, glucose, lactic acid, and ATP levels were analyzed to investigate the impact of PGM2L1 on glycolysis. Our findings revealed that overexpressing PGM2L1 in TNBC cells under hypoxia led to significant increases in glucose consumption, lactic acid production, and ATP synthesis. Conversely, the opposite effect was observed in the PGM2L1 knockdown group (Fig. 4F–H). These results indicated that PGM2L1 effectively elevated the glycolytic activity in TNBC cells under hypoxic conditions. Considering that the primary function of PGM is to catalyze the interconversion between G-1-P and G-6-P, we evaluated the G-6-P levels in TNBC cells. The results demonstrated that G-6-P levels increased in TNBC cells overexpressing PGM2L1, while they declined in groups with PGM2L1 silencing (Fig. 4I). Taken together, these findings confirmed that PGM2L1 facilitated the glycolysis in TNBC cells by enhancing the conversion from G-1-P to G-6-P under hypoxic conditions.

To further validate whether the biological impact of CAF-secreted CSF3 on TNBC cells was mediated by PGM2L1, we treated the TNBC cells transiently transfected with PGM2L1 siRNA or NC with hypoxic CAF-CM. Glucose consumption, lactic acid, and ATP generation, and G-6-P levels were found to decrease in the PGM2L1 knock-down group (Fig. 5A–D). Subsequently, 2-Deoxy-D-glucose (2-DG), a non-metabolized glucose analog, was used to inhibit glycolysis. We found that 2-DG significantly abolished PGM2L1-mediated enhancement of TNBC cells proliferation, further confirming that PGM2L1 promoted tumor cell progression through enhanced glycolysis (Fig. 5E).

As evidenced by MTT and EdU assays, silencing PGM2L1 impeded the proliferative capacity of MDA-MB-231 and MDA-MB-468 cells treated with hypoxic CAF-CM (Fig. 5F, Fig. 5G and Supplementary Fig. 5G). Transwell assays revealed that PGM2L1 silencing was sufficient to inhibit the migration and invasion of TNBC cells treated with hypoxic CAF-CM (Fig. 5H). Western blot analysis of EMT markers also indicated that PGM2L1 knockdown led to decreased levels of FN1 and N-cad and increased expression of E-cad (Fig. 5I). Collectively, the data above demonstrated that PGM2L1 enhanced TNBC cells proliferation, migration and invasion by boosting glycolysis and generating more energy, such as ATP, to support tumor progression.

### Down-regulation CSF3R inhibits PGM2L1-mediated TNBC progression and glycolytic reprogramming

**Fig. 6:**
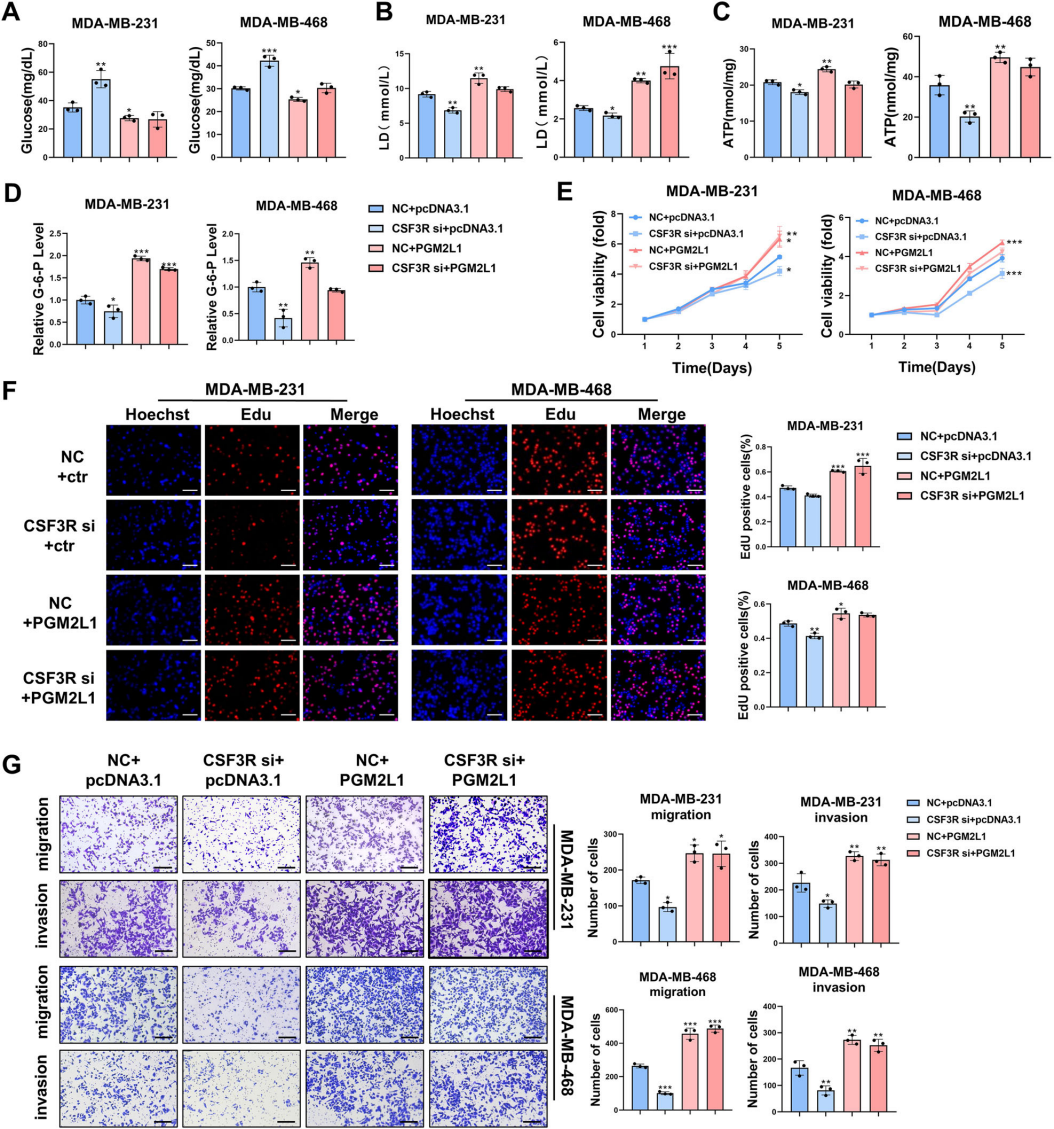
Down-regulation CSF3R inhibits PGM2L1-mediated TNBC progression and glycolytic reprogramming. Following CSF3R-siRNA and PGM2L1-ove plasmid transfection, TNBC cells were tested by glycolysis level, including glucose (**A**), lactic acid (**B**), ATP (**C**), and G-6-P (**D**) (n = 3). MTT(**E**, n = 4) and EdU (**F**, n = 3) assays were performed to assessed cellular proliferation ability. Scale bars, 100 μm. **G** Transwell assay was employed to appraise cells migration and invasion abilities (n = 3). Scale bars, 200 μm. The data are presented as the mean ± SD. *P < 0.05, **P < 0.01, ***P < 0.001.

To confirm that the CSF3/CSF3R axis enhances TNBC progression under hypoxia primarily through PGM2L1, rescue assays were performed. MDA-MB-231 and MDA-MB-468 cells were co-transfected with CSF3R-siRNA and PGM2L1 overexpression plasmid or their respective negative controls. Glycolysis indexes of these cells were then tested using colorimetric assays. The results demonstrated that overexpression of PGM2L1 restored glycolysis levels in TNBC cells under hypoxic conditions (Fig. 6A–D).

Next, MTT and EdU assays illustrated that knockdown of CSF3R attenuated the proliferation ability of TNBC cells, and this effect could be reversed by PGM2L1 overexpression (Fig. 6E, F). Moreover, the migration and invasion abilities of TNBC cells were significantly augmented in the groups co-transfected with CSF3R-siRNA and PGM2L1 overexpression plasmids compared to those with only CSF3R-siRNA (Fig. 6G). These findings confirmed that the CSF3/CSF3R axis promoted the malignant behaviors of TNBC cells through regulating PGM2L1.

### CSF3 and PGM2L1 promote TNBC progression in vivo

**Fig. 7:**
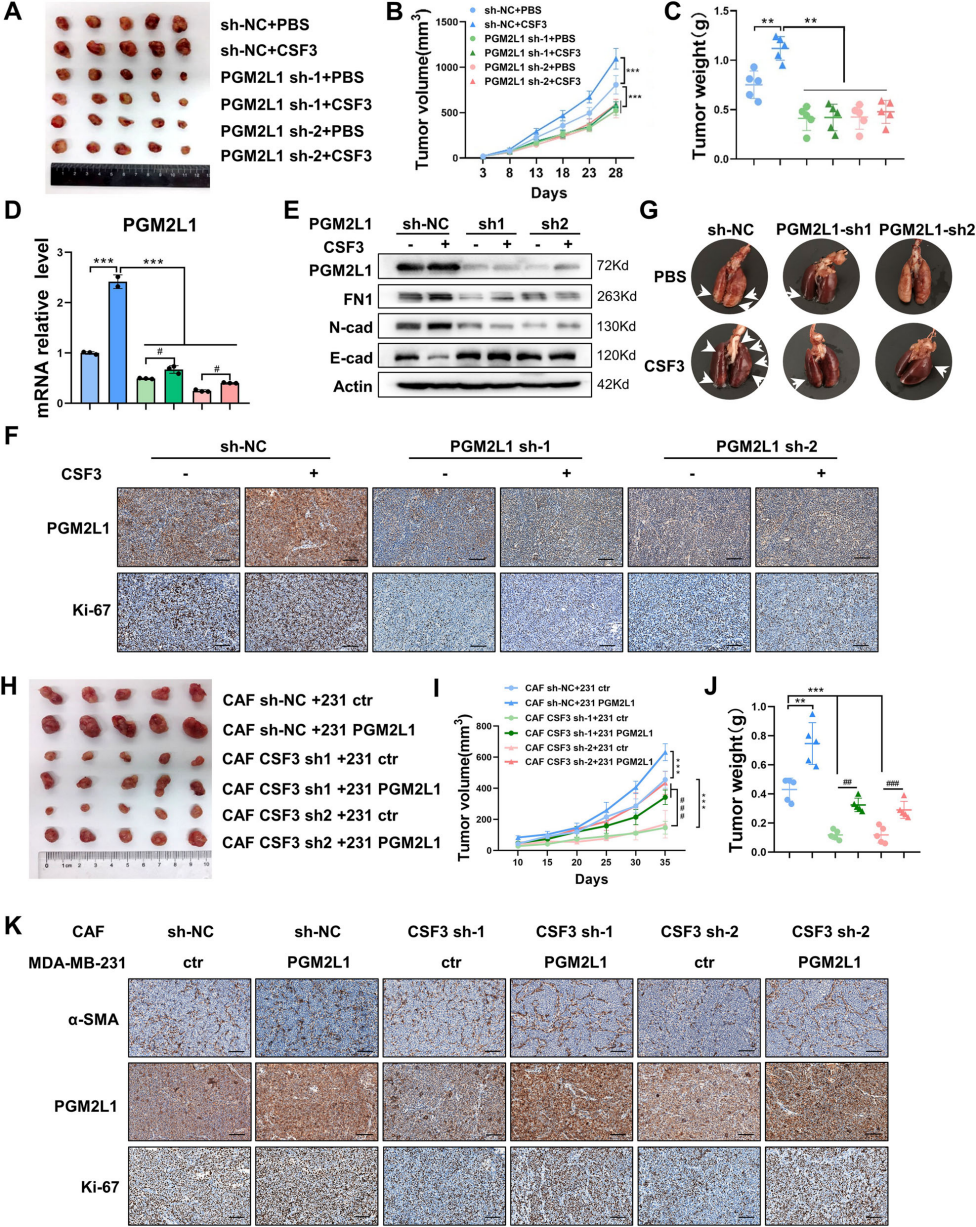
CSF3 and PGM2L1 promote TNBC progression in vivo. **A** Control or PGM2L1-knockdown MDA-MB-231 cells were transplanted into nude mice, followed by PBS or CSF3 treatment. Images of tumors were harvested (n = 5 mice). **B** Tumor volumes were measured once 5 days beginning 3rd day after implantation (n = 5 mice). **C** Tumor weight was measured in each group (n = 5 mice). **D** The expression of PGM2L1 of xenograft tumors were examined by qRT-PCR (n = 3). **E** The protein level of PGM2L1 and EMT-related markers of xenograft tumors were examined by western blot. **F** Ki-67 and PGM2L1 in each group were tested by IHC staining. Scale bars, 100 μm. **G** Representative lung metastatic nodules in mice from different groups were counted (n = 5 mice). **H** Images of xenograft tumors of MDA-MB-231 cells mixed CAFs (n = 5 mice). **I** Tumor volumes were measured once 5 days beginning 10th day after implantation (n = 5 mice). **J** Tumor weight was measured in each group (n = 5 mice). **K** IHC staining of α-SMA, Ki-67, and PGM2L1 in each group (n = 5). Scale bars, 100 μm. The data are presented as the mean ± SD. ^#^*P* < 0.05, **^,##^*P* < 0.01, ***^,###^*P* < 0.001.

To elucidate the in vivo roles of CSF3 and PGM2L1, MDA-MB-231 cells with PGM2L1 silenced were subcutaneously implanted into female Balb/c-Nude mice allocated randomly. Concurrently, PBS or CSF3 (500 ng/mouse) was administered through the tail vein weekly. After 4 weeks, the mice were anesthetized and sacrificed. The results displayed that growth rates and tumor weights of xenograft tumors in the sh-NC + CSF3 group were higher than in the control group, while the knockdown of PGM2L1 abolished this trend (Fig. 7A–C). Further, qRT-PCR and western blot analyses revealed that PGM2L1 expression was higher in the sh-NC + CSF3 group compared to the sh-NC + PBS group (Figs. 7D, E). Furthermore, the level of E-cad was augmented in sh-PGM2L1 groups, wherase the expression of FN1 and N-cad displayed the opposite change, indicating that PGM2L1 promotes EMT in vivo (Fig. 7E). IHC analysis further revealed that Ki-67 expression was elevated in the sh-NC + CSF3 group and reduced in the sh-PGM2L1 groups compared to the control group (Fig. 7F). These findings indicated that CSF3 enhanced xenograft tumor growth through PGM2L1 mediation in vivo.

To assess tumor invasiveness, MDA-MB-231 cells with PGM2L1 knockdown were injected into the tail vein of Balb/c-Nude mice and then PBS or CSF3 was administered weekly. After 7 weeks, lung metastases were evaluated. The sh-PGM2L1 + PBS group displayed the fewest pulmonary metastatic foci, while the sh-NC + CSF3 group had the highest number of lung tumor nodules (Fig. 7G and Supplementary Fig. 7A). H&E staining showed that the sh-NC + CSF3 group had a greater number and larger size of metastatic nodules, whereas the sh-PGM2L1 group had the smallest metastatic nodules (Supplementary Fig. 7B).

In addition, we utilized lentiviral vectors to successfully produce CSF3-knockdown CAFs and PGM2L1-overexpressing MDA-MB-231 cells. These engineered MDA-MB-231 cells were then mixed with either sh-NC CAFs or sh-CSF3 CAFs and subcutaneously transplanted into female Balb/c-Nude mice allocated randomly. After 5 weeks, the mice were sacrificed to assess the tumor volume and weight. Mice injected with the mixture of MDA-MB-231 cells and sh-CSF3 CAFs developed significantly smaller tumors compared to those injected with MDA-MB-231 cells and sh-NC CAFs. However, the reduction in tumor volume observed in sh-CSF3 CAFs groups could be mitigated by the presence of PGM2L1-overexpressing MDA-MB-231 cells (Fig. 7H–J). Moreover, the protein levels of PGM2L1 were tested in different groups via western blot (Supplementary Fig. 7C). IHC assays were executed to assess the expression of relative molecules. α-SMA was detected in all groups to confirm the presence of CAFs in tumors. Enhanced Ki-67 expression was detected in PGM2L1 overexpressing groups, further indicating that PGM2L1 facilitated the growth of tumors in vivo (Fig. 7K). These findings supported the conclusion that CSF3 secreted by CAFs enhanced tumor growth by regulating PGM2L1 in vivo. Taken together, our data verified that CAF-derived CSF3 plays a crucial role in regulating tumor growth and metastasis through PGM2L1 in TNBC.

## Discussion

Previous studies have highlighted the reciprocal relationships between BC cells and surrounding CAFs [4]. However, most research on CAF-tumor cell interactions focus on normoxic conditions [20, 21], overlooking the hypoxic environment characteristic, which is more pronounced in TNBC compared to other BC types. Under hypoxic conditions, a series of relevant signaling pathways are activated to increase cancer cells invasiveness and metabolic reprogramming for cell survival [5]. Besides changes in tumor cells, other cellular components of the TME also influence the malignant phenotype in response to hypoxic conditions [22]. The specific interactions between CAFs and tumor cells under hypoxic conitions remain underexplored. A study using mouse models of hypoxic-ischemic brain injury showed that CSF3 significantly increases HIF-1α expression in astroglia cells during acute hypoxia [23]. Present study is the first to identify the transcriptional regulation of HIF-1α on CSF3 in CAFs, providing further evidence of the close relationship between HIF-1α and CSF3. Currently, it is established that CSF3 promotes growth, metastasis, angiogenesis, and modulates anti-tumor immunity in various solid tumors [24]. Neutrophils and cancer-associated adipocytes (CAAs) can secrete CSF3 [25, 26]. which acts through autocrine or paracrine signaling to enhance BC malignancy. This study identified CAFs as a novel source of CSF3 in the TME and proved that CSF3, derived from CAFs, promotes the progression of TNBC cells.

Previous studies have reported that the pro-tumor effects of CSF3 are primarily mediated by neutrophils and myeloid-derived suppressor cells(MDSCs), which are the main populations expressing CSF3R. The MDSCs can be recruited by CSF3 secreted by tumor cells under hypoxic conditions and participate in the formation of pre-metastatic niches in the lungs [27]. The mechanism has gradually been revealed: CSF3 promotes BC lung metastasis by mobilizing Ly6G^+^Ly6C^+^ granulocytes to the lungs, creating a pre-metastatic lung microenvironment [28, 29]. In contrast, this study demonstrates a different mechanism in which CSF3 directly regulates tumor cells rather than acting on MDSCs. Our data suggest that CSF3 secreted by hypoxic CAFs in the primary tumor not only directly affects the proliferation and metastasis of adjacent tumor cells through paracrine signaling but also promotes the formation of pre-metastatic niches in the lung by entering the bloodstream, thereby exerting a dual impact on tumor cell invasion and distant colonization.

Metabolic reprogramming is one of the hallmarks of cancer, enabling tumor cells to adjust their metabolic pathways according to environmental conditions to meet their bioenergetics needs and support oncogenic progression [30, 31]. In cancer cells, enhanced glycolysis generates additional carbon intermediates and precursor molecules for biosynthesis and other metabolic pathways, thereby promoting the anabolic metabolism required for cell proliferation. This shift diminishes the reliance on the tricarboxylic acid (TCA) cycle, a phenomenon commonly referred to as the Warburg effect [30, 32]. Previous studies have demonstrated that both CAFs and cancer cells can function as producers or recipients in the exchange of metabolites, with both metabolites and cytokines serving as mediators of metabolic regulation [33–35]. This suggests a complex metabolic regulatory mechanism between tumor cells and CAFs, requiring further investigation. Furthermore, substantial evidence indicates that hypoxia influences tumor metabolic reprogramming, leading to neoangiogenesis and metastasis [36–38]. In the present study, we explored the mechanism of metabolic reprogramming between CAFs and tumor cells under simulated hypoxic conditions. It was found that CAFs upregulated the PGM2L1 in TNBC cells by secreting CSF3 under hypoxia, and then the enhanced glycolysis in tumor cells promoted progression and adapted to the hypoxic microenvironment. This finding provides novel insights into the metabolic reprogramming between cellular components in the hypoxic TME.

Recent research on the PGM family has primarily focused on their role in prognostic analysis. It has been reported that patients with high expression levels of PGM1, PGM2, and PGM5 have better prognoses [39]. A risk score prediction model based on five glycolysis-related genes has also confirmed that PGM2L1 is associated with a poor prognosis in prostate cancer [19]. PGM family members are crucial in modulating the conversion between G-6-P and G-1-P, enabling the reversible regulation between glycolysis and glycogen synthesis. However, their regulatory roles in glucose metabolism are not consistent. Guang-Zhi Jin et al. reported that PGM1 suppresses liver cancer progression by promoting glycogen synthesis and inhibiting glycolysis [40]. Interestingly, a 2019 study revealed that PGM1 facilitates the conversion of G-1-P to G-6-P, thereby increasing glycolysis [33]. In general, the roles of PGM in regulating glucose metabolism in tumors and the underlying mechanisms have not yet been elucidated, requiring further investigation. The present study explored the function of PGM2L1 in hypoxic environment for the first time, contributing to the evidence of PGM2L1’s involvement in glucose metabolism under hypoxia. Our findings revealed that PGM2L1 promotes the conversion of G-1-P (glycogen synthesis) to G-6-P (glycolysis) under hypoxic conditions, which is consistent with the metabolic tendency of tumor cells to favor glycolysis over the TCA cycle, thereby providing energy to sustain their malignant phenotype. Importantly, the cancer-promoting effect of PGM2L1 can be partially counteracted by the glycolysis inhibitor 2-DG. This study is also the first to demonstrate in vitro and in vivo that PGM2L1 regulates EMT under hypoxic conditions, supporting previous evidence that PGM2L1 promotes tumor cell metastasis [19]. In addition, this study explored the clinical characteristics of PGM2L1 in TNBC samples for the first time, indicating that PGM2L1 is relevant to TNBC oncogenic progression.

**Fig. 8:**
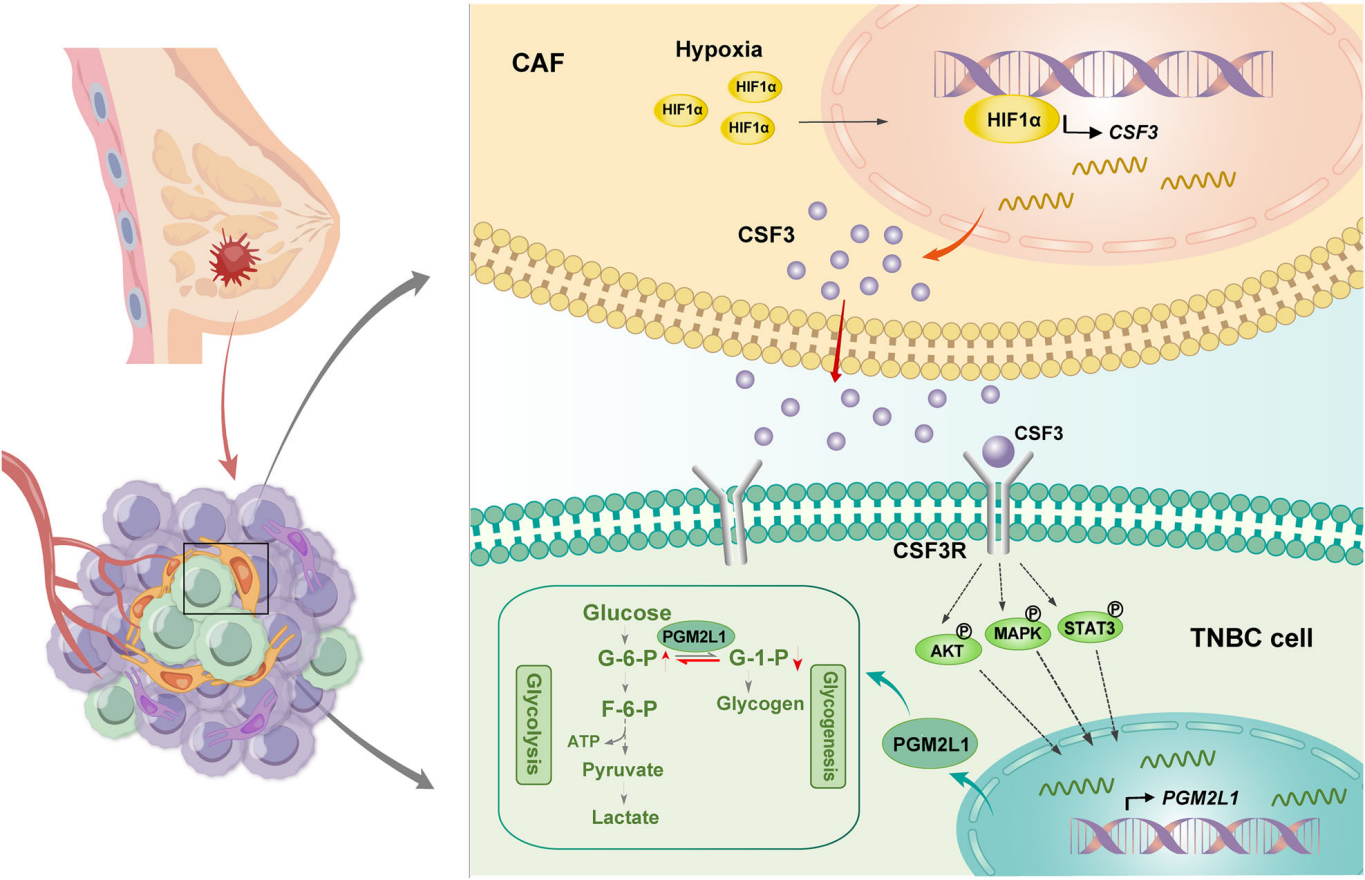
A schematic graph of the mechanism. CSF3 acts as a key mediator integrating CAFs and TNBC cells, promoting TNBC proliferation and metastasis via enhancing PGM2L1-dependent glycolysis reprogramming.

In conclusion, this study discovered that hypoxia signal elevated CSF3 expression in CAFs by activating the HIF-1α transcriptional activity. CSF3 secreted by CAFs acted on CSF3R of adjacent TNBC cells, further augmenting the expression of PGM2L1. In cancer cells, PGM2L1 upregulated the level of glycolysis by promoting the conversion from G-1-P to G-6-P, providing energy for tumor progression and metastasis (Fig. 8). These findings offer new insights into the mechanisms by which CAFs promote malignant progression in BC under hypoxia, and suggest that targeting PGM2L1 may represent a promising strategy to suppress BC growth and metastasis. However, our study has several limitations. First, although we have demonstrated that the expression of CSF3R is associated with hypoxic conditions, the specific mechanism remains unclear and requires further investigation. Second, due to the heterogeneity of CAFs, the CSF3 secretion levels vary greatly among different CAFs. In future research, it is necessary to conduct a more in-depth analysis of the CAF subtypes with higher CSF3 secretion levels.

## Materials and methods

### Cell cultures

The paired NFs and CAFs from TNBC patients were cultured as previously described [41]. Briefly, CAFs were extracted from BC tissues, and NFs were obtained from adjacent normal tissues. Tissue samples were digested for 2 h at 37 °C with collagenase (100 μg/mL, #07902; Stem Cell Technologies, Seattle, WA, USA) and hyaluronidase (100 μg/mL, #07912; Stem Cell Technologies) in Dulbecco’s modified Eagle’s medium (DMEM; HyClone, Logan, UT, USA) supplemented with 10% fetal bovine serum (FBS, S711-001S; LONSERA, Shanghai, China). After digestion, the samples were centrifuged, and the resulting cell pellets were resuspended and filtered through a 100 μm strainer. The filtered cells were plated in 60 mm culture dishes and cultured in DMEM with 10% FBS, penicillin (100 U/mL), and streptomycin (100 μg/mL) at 37 °C in a humidified 5% CO_2_ atmosphere. The paired NFs and CAFs were further identified by the expression of CAF markers, including α-SMA, FAP, FSP1, and vimentin.

The human TNBC cell lines MDA-MB-231, MDA-MB-468, and 293T cells were purchased from American Type Culture Collection (Manassas, VA, USA) and were cultured in DMEM with 10% FBS, 100 U/mL penicillin, and 100 μg/mL streptomycin at 37 °C in a humidified incubator with 5% CO_2_ under normoxic (20% O_2_) or hypoxic (1% O_2_) conditions. Short tandem repeat (STR) profiling was used to authenticate these cell lines, and it was confirmed that there was no mycoplasma contamination.

### Plasmid, small interfering RNA (siRNA), and Transfection

The pcDNA3.1 vector carrying homo-PGM2L1-Flag plasmid was purchased from BioSune (Shanghai, China). pCMV-HIF1ɑ-Flag plasmid was obtained from MiaoLingBio (Wuhan, China). Lentivirus-mediated shRNA targeting CSF3 was designed and synthesized by Keyybio (Jinan, China). HIF-1α siRNA, CSF3R siRNA, PGM2L1 siRNA, and negative control were obtained from GenePharma (Suzhou, China). All RNAi sequences are shown in Table 1.

**Table 1:** siRNA used for transfection.

Name	Sense (5’-3’)	Anti-sense (5’-3’)
HIF-1α si	AGUCACCACAGGACAGUAUTT	AUACUGUCCUGUGGUGACUTT
CSF3R si-1	GGACCCAGGAAUCUAUCAUTT	AUGAUAGAUUCCUGGGUCCTT
CSF3R si-2	GACGGAUCCAAGGUUAUGUTT	ACAUAACCUUGGAUCCGUCTT
PGM2L1 si-1	GAACAGAACCAAAGAUAAATT	UUUAUCUUUGGUUCUGUUCTT
PGM2L1 si-2	GGAGAAUGGUUGUUGGAAATT	UUUCCAACAACCAUUCUCCTT
NC	UUCUCCGAACGUGUCACGUTT	ACGUGACACGUUCGGAGAATT

JetPRIME (PolyPlus, Strasbourg, France) was employed to transfect cells with plasmid based on provided directions. siRNA transfection was performed using RFect (Baidai, Changzhou, China) according to the manufacturer’s instructions.

### RNA extraction and quantitative real-time PCR (qRT-PCR)

Total RNA was extracted from cells or tissues using a Total RNA Extraction reagent (R701, Vazyme, Nanjing, China) according to the manufacturer’s instructions. Then complementary DNA (cDNA) was synthesized using Evo M-MLV RT Premix for qRT-PCR (AG11706; Accurate Biology, Changsha, China). The qRT-PCR was performed with SYBR Green PCR Master Mix (RR064A; Takara, Dalian, China). The relative mRNA expression levels were normalized to β-actin and calculated using the 2^-ΔΔCT^ method. The primers used are listed in Table 2.

**Table 2:** Primer sequences used for qRT-PCR.

Genes	Forward sequence (5’-3’)	Reverse sequence (5’-3’)
β-actin	CACTGTGCCCATCTACGAG	AATGTCACGCACGATTTCC
CSF3	CGACTTTGCCACCACCATCT	GTAGAACGCGGTACGACACC
HIF-1α	TATGAGCCAGAAGAACTTTTAGGC	CACCTCTTTTGGCAAGCATCCTG
CSF3R	CTTGTGGCCTATAACTCAGCC	CCCACTCAATCACATAGCCCT
PGM2L1	TTACTGCCTCTCACAACCGC	GCGGGCTGGTATCCACTAAA
DPYD	CGGCGGACATCGAGAGTAT	TCCTCGCTCACCAAGAGTCG
GALNT4	TGGCCTATATCTTCGTGGAGC	CTGGAGTTTGCTGGCTTTCC
GLCE	TGAAACAGCAGAAGACAGAGACA	GCCACATTCGCCATAAGCAC
ATP6V1A	TGGAGGGTGACATGGCTACT	GAGGGGTTTACCAGTGCGAA
INPP4B	TGGCTGGCTGCAACGATTT	CTCTTCTCATGCAATCCAGCG
PLD1	AAAATCTGGACACGCGGGAA	GACAGCCGGAGAGATACGTC
IPMK	TCAGCAACAGGTCAGCAAGT	AGCTTCTTCCGTAATGCTGGT
MECOM	TCCGGTACAGCAACATCGTC	TGCCATTCATTCTCTCCTCCAC
GALNT5	TTGGAACATACGACCCTGGC	CCACCACACATCCACACCTT
PFKL	GGGAGGTGAGAACATCAAGCA	CCAATGATAGTGCCGCCCAG
ENO1	AACCCAAAGAGGATCGCCAA	CCCGAACGATGAGACACCAT
PKM1F	CAGCACCTGATAGCTCGTGA	TGAGGCTCGCACAAGTTCT
PDK1	GCCACTATGGAACACCATGC	CCTCATTACCCAGCGTGACA
HK2	GCCCGCCAGAAGACATTAGA	GCTCAGACCTCGCTCCATTT
PKM2	AATCACGCTGGATAACGCCT	TCGGCACCTTTCTGCTTCAC

### Western blot analysis

Cells were harvested with lysis buffer (P0013; Beyotime, Shanghai, China) containing protease inhibitors PMSF (1:100), and then subjected to sodium dodecyl sulfate-polyacrylamide gel electrophoresis (SDS-PAGE) gel according to the concentration of protein. After seprating in the gels, proteins were transferred onto polyvinylidene fluoride (PVDF) membranes (Millipore, MA, USA). Blots were blocked using 5% non-fat milk and then incubated with the primary antibodies over night at 4 °C. After washing, the blots were incubated with HRP-conjugated secondary antibodies (1:3000; ZSGB-Bio, Beijing, China) at room temperature for at least 1 h. Protein bands were detected using the SuperPico ECL Chemiluminescence Kit (E422; Vazyme). β-actin served as a loading control. The primary antibodies used in this study are shown in Table 3.

**Table 3:** Antibodies used for western blot and IHC.

Name	Supplier	Catalog#	Application
α-SMA	Proteintech^a^	55135-1-AP	WB (1:500) IHC (1:300)
FAP	Affinity^b^	AF5344	WB (1:500)
FSP1	Abclonal^c^	A1631	WB (1:500)
FN1	Proteintech	15613-1-AP	WB (1:500)
Vimentin	Servicebio^d^	GB11192	WB (1:500)
CSF3R	Proteintech	18310-1-AP	WB (1:200)
p-ERK	Affinity	AF1015	WB (1:1000)
ERK	Affinity	AF0155	WB (1:1000)
p-AKT	Affinity	AF0016	WB (1:500)
AKT	PTM BIO ^e^	PTM-5191	WB (1:500)
p-STAT3	Affinity	AF3293	WB (1:500)
STAT3	Affinity	AF6294	WB (1:500)
N-cadherin	Proteintech	22018-1-AP	WB (1:500)
E-cadherin	Proteintech	20874-1-AP	WB (1:500)
PGM2L1	Proteintech	13942-1-AP	WB (1:500) IHC (1:100)
Ki-67	Proteintech	28074-1-AP	IHC (1:500)
Actin	Affinity	T0022	WB (1:1000)

^a^Proteintech, Chicago, IL, USA.

^b^Affinity, Changzhou, China.

^c^Abclonal, Wuhan, China.

^d^Servicebio, Wuhan, China.

^e^PTM BIO, Hangzhou, China.

### Cell proliferation assay

TNBC cells were seeded in 96-well plates and cultured for the indicated time periods. Then, 20 µl 3-(4,5-dimethylthiazol-2-yl)-2,5-diphenyltetrazolium bromide (MTT) (M8180; Solarbio, Beijing, China) was added to each well for 4-6 h at 37 °C. After aspiration of the supernatants, 100 µl dimethyl sulfoxide (DMSO) was added. The absorbance was measured in microplate reader (ZEISS, Oberkochen, Germany).

### Ethynyldeoxyuridine (EdU) assay

An EdU assessment kit (Ribobio, Guangzhou, China) was employed for the assessment of cellular multiplication. Briefly, cells were seeded in 96-well plates (1 × 10^4^/well). After incubation for 2 h with EdU buffer (50 μM) at 37 °C, the cells were fixed for 30 minutes (min) with 4% paraformaldehyde and stained with Apollo dye for 1 h. The nucleus were stained with Hoechst for 30 min. The EdU incorporation rate was calculated as the ratio of the number of Edu-incorporated cells to the number of Hoechst-staining cells.

### Migration and invasion assay

Migration and invasion assays were performed using the transwell system (pore size 8 μm; Corning Costar, Lowell, MA, USA). For the migration assay, 8 × 10^4^ cells in 200 μL of serum-free DMEM were seeded in the upper chamber of a transwell system, while 700 μL of DMEM containing 20% FBS, CAF-CM, or CAFs was added to the lower chamber. After incubation for the specified time, cells that adhered to the lower surface of the membrane were visualized using a light microscope and counted for statistical analysis. In the invasion assay, transwell inserts coated with Matrigel (356234; Corning Costar) were used.

### ELISA assay

The CSF3 concentration in the culture supernatant of cells was measured by a human CSF3 ELISA kit (1117312; Dakewe, Shenzhen, China) according to the manufacturer’s protocol. Briefly, the culture medium was collected and centrifuged at 2000 rpm. A 100 µL aliquot of the supernatant was added to an ELISA plate and incubated with a biotin-labeled CSF3 antibody. After washing the plate three times with washing buffer, 100 µL of HRP-conjugated streptavidin solution was added to each well and incubated for 45 min at room temperature. Following this, the plate was incubated with 100 µL of TMB reagent for 30 min at room temperature. Finally, 100 µL of stop solution was added, and the absorbance was immediately measured at 450 nm using a microplate reader (ZEISS).

### Dual-luciferase reporter gene assay

The wild-type or mutant CSF3 5’ UTR, containing potential HIF-1α binding sites, was amplified by PCR and inserted into the pGL4.26 vector. Cells were then co-transfected with the luciferase plasmids, HIF-1α, and reporter vectors. After 48 h of incubation, dual-luciferase activity was measured using the Dual-Luciferase Reporter Assay Kit (Promega, Madison, WI, USA) according to the manufacturer’s instructions. Renilla luciferase activity was used for normalization.

### Immunofluorescence (IF) staining

Cells on slides were fixed with 4% paraformaldehyde for 15 min. After washing with PBS, the cells were permeabilized with 0.2% Triton X-100 and then blocked with 10% goat serum. Next, the cells were incubated with primary antibodies at 4 °C overnight. After washing, the cells were incubated with corresponding secondary fluorescent antibodies for 2 h at room temperature, and protected from light. DAPI (C1005, Beyotime) was then used for nuclear counterstaining. Finally, images were obtained by a fluorescent microscope (ZEISS).

### In vivo proliferation and metastasis assay

Female Balb/c-Nude mice (4–6 weeks old) were purchased from GemPharmatech (Nanjing, China) and housed in the Laboratory Animal Center at Shandong University. Mice were allocated randomly into six groups and five mice were used in every group. For proliferation analysis, 1 × 10^7^ MDA-MB-231 cells or MDA-MB-231 cells mixed with CAFs at a 1:2 ratio were suspended in 100 µL PBS and basement membrane matrix (356234; Corning) and injected subcutaneously into the mice. Tumor growth was monitored every 5 days using vernier calipers to measure the long and short diameters of the tumors. Tumor volume was calculated using the formula: volume = 0.5 × length × width². The mice were euthanized, and tumor weight and volume were examined. For the metastasis model, 5 × 10^5^ MDA-MB-231 cells were injected into the tail veins of female Balb/c-Nude mice (4–6 weeks old). After 7 weeks, lungs were collected for nodule counting and H&E staining. Investigators who conducted animal studies were blinded to the experimental details.

### Immunohistochemical (IHC) staining

Tumor tissues were fixed in 10% neutral formalin. After dehydration and clearing, the tissues were embedded in paraffin and sectioned into 6 µm slices. The IHC was conducted using a ZSGB-Bio kit (SP9000) according to the manufacturer’s instructions. Following dewaxing and antigen retrieval in sodium citrate buffer, the sections were blocked with 5% BSA and incubated with primary antibodies overnight at 4 °C. The sections were then treated with peroxidase-conjugated secondary antibodies for 30 min at room temperature. After staining with diaminobenzidine (DAB) and counterstaining with Mayer’s hematoxylin. Finally, the sections were examined under a microscope (Leica, Wetzlar, Germany).

### Hematoxylin and eosin (H&E) staining

Lung tissues were embedded in 10% neutral formalin and sectioned into 6 µm slices. After dewaxing through a series of ethanol solutions with increasing concentrations, the slides were sequentially stained with hematoxylin and eosin. Finally, the sections were examined under a microscope (Leica).

### Measurement of glucose, ATP, lactic acid, and glucose-6-phosphate (G-6-P)

Glucose levels were measured using the Glucose Colorimetric Detection Kit (EIAGLUC; Invitrogen, Carlsbad, CA, USA). ATP and G-6-P levels were assessed using the Enhanced ATP Assay Kit (S0027; Beyotime) and the G6P Assay Kit with WST-8 (S0185; Beyotime), respectively. Lactic acid was measured with the Lactic Acid Assay Kit (A019-2-1; Jiancheng Bio, Nanjing, China). All assays were performed according to the manufacturer’s instructions and results were obtained using a microplate reader (ZEISS).

### Reagents

Other reagents used in the experiment are shown in Table 4.

**Table 4:** Reagents used in the experiment.

Name	Supplier	Catalog	Application
CSF3	R&D System^a^	214-CS	50 ng/ml
2-DG	Med Chem Express^b^	HY-13966	500 μM
ERK inhibitor	Med Chem Express	SCH772984	2.5 μM
AKT inhibitor	Med Chem Express	MK-2206	5 μM
Stat3 inhibitor	Med Chem Express	NSC 74859	5 μM

^a^R&D System, Minneapolis, MN, USA.

^b^Med Chem Express, Rahway, NJ, USA.

### Statistics analysis

Results were analyzed by GraphPad Prism 9.0 (USA) and are expressed as the mean ± standard deviation (SD). Comparisons between two groups were performed using either a two-tailed non-parametric or parametric Student’s t-test, depending on the data distribution. For multiple groups, one-way or two-way analysis of variance (ANOVA) was used. Survival analysis was conducted using the Kaplan-Meier method and evaluated by the log-rank test. The experiments were repeated at least three times. *P* < 0.05 was considered statistically significant.

## Supplementary information

Supplemental Material

Western blots

## Data Availability

The data used or analyzed during the current study are available from the corresponding author on reasonable request.
