# Supplementary material for: Cancer-associated fibroblasts secrete CSF3 to promote TNBC progression via enhancing PGM2L1-dependent glycolysis reprogramming
Source: Cell Death Dis. 2025 Apr 4;16(1):249. doi: 10.1038/s41419-025-07580-6 (PMC11971334; doi:10.1038/s41419-025-07580-6)
Supplement: Supplementary file 1 — Supplemental Material [file 41419_2025_7580_MOESM1_ESM.pdf]

Supplementary materials

Cancer-associated fibroblasts secrete CSF3 to promote TNBC progression via enhancing PGM2L1-dependent glycolysis reprogramming

Wenqi Qin[1*], Bing Chen[2*], Xin Li[1], Wenjing Zhao[2], Lijuan Wang[2], Ning Zhang[1], Xiaolong Wang[1], Dan Luo[1], Yiran Liang[1], Yaming Li[1], Xi Chen[1], Tong Chen[1] and Qifeng Yang[1,2,3#]

Affiliations

[1] Department of Breast Surgery, General Surgery, Qilu Hospital of Shandong University, Wenhua Xi Road No. 107, Jinan, Shandong, 250012, P.R. China.

[2] Biological Resource Center, Qilu Hospital of Shandong University, Jinan, Shandong, 250012, P.R. China.

[3] Research Institute of Breast Cancer, Shandong University, Jinan, Shandong, 250012, P.R. China.

[#] Correspondence: Qifeng Yang, E-mail: qifengy_sdu@163.com

[*] Wenqi Qin and Bing Chen contributed equally to this work.

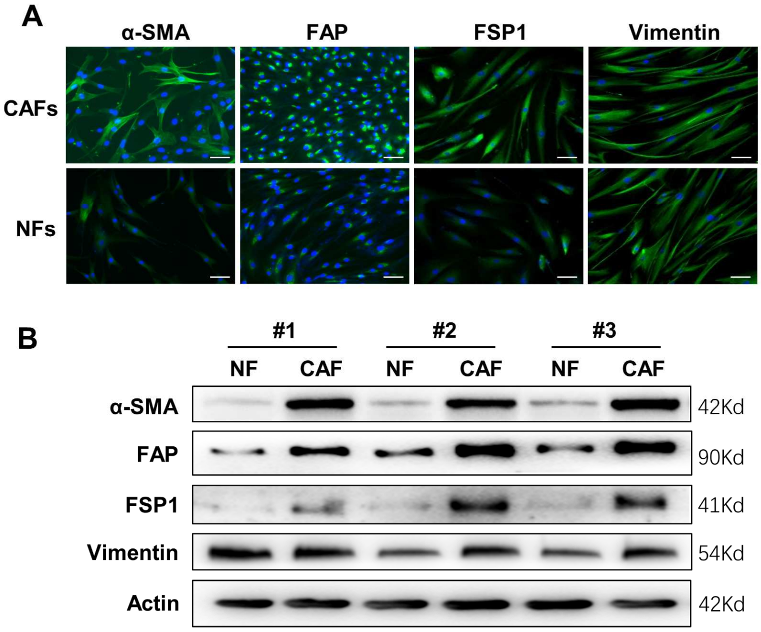

Fig S1. The characteristics of paired NFs and CAFs were identified.

(A, B) Immunofluorescent staining (A) and western blot (B) of α-SMA, FAP, FSP1 and vimentin expression in NFs and CAFs. Scale bars, 100 μm.

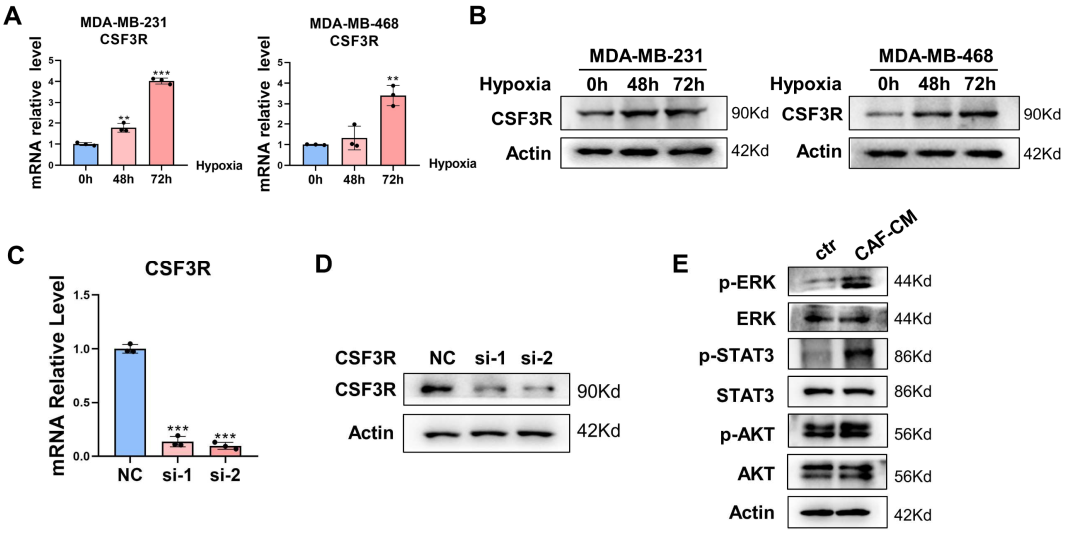

Fig S2. CSF3R is upregulated under hypoxic conditions.

(A, B) The mRNA and protein level of CSF3R in TNBC cells increased with persistent. (n=3). (C, D) CSF3R was effectively silenced in TNBC cells by transfecting siRNA (n=3). (E) ERK, STAT3 and AKT pathways in MDA-MB-231 cells were activated by hypoxic CAF-CM. The data are presented as the mean ± SD. ** P <0.01, *** P < 0.001.

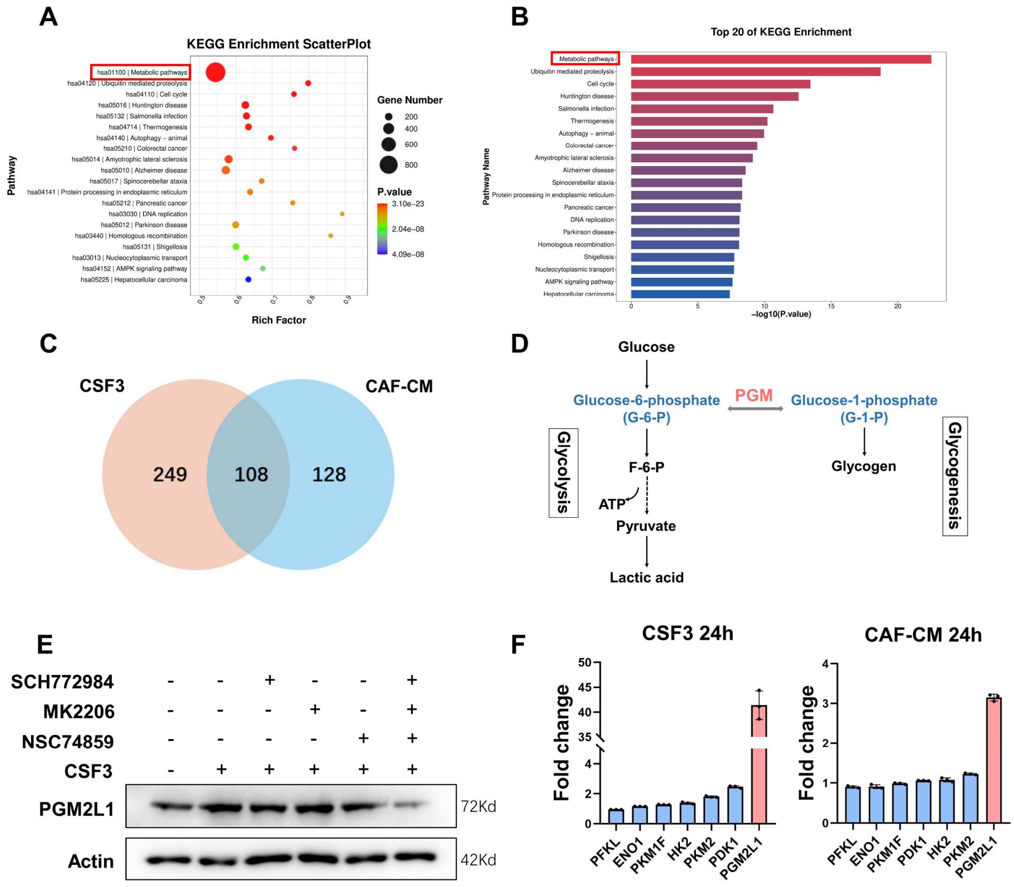

Fig S3. CSF3 increases PGM2L1 in TNBC cells.

A, B Top 20 KEGG enrichment pathway of the RNA-Seq data. (Fold change of > 1.5, *P* value < 0.05). **C** Venn diagram of upregulated metabolic-related genes in both CSF3 and CAF-CM groups. **D** Diagram of PGM regulating glucose metabolism. **E** The protein level of PGM2L1 was influenced by pathway inhibitors. **F** The mRNA expression level of key glycolysis-related genes by qRT-PCR (n=3). The data are presented as the mean ± SD.

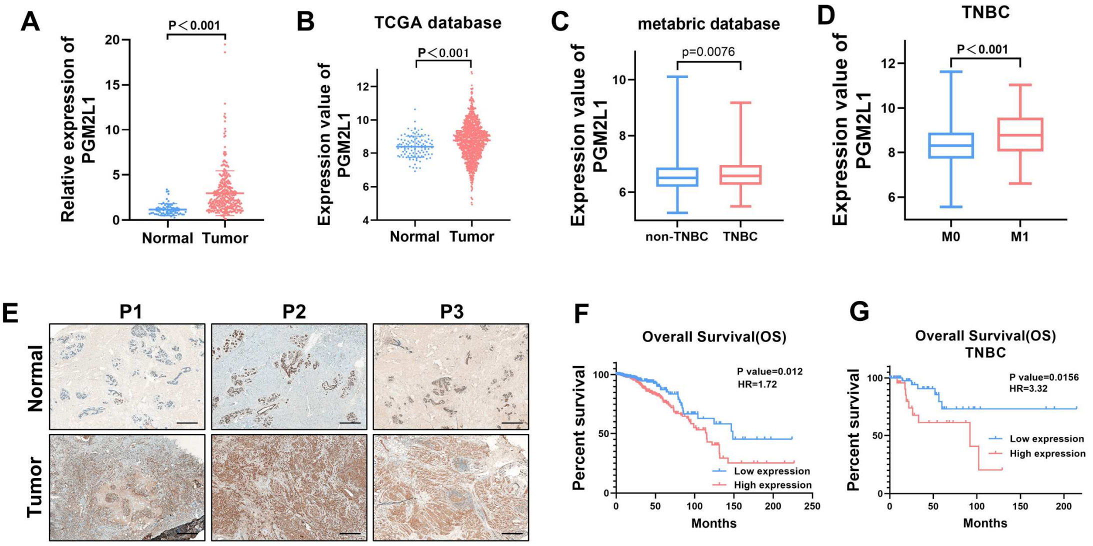

Fig S4. Clinical significance of PGM2L1 in breast cancer.

A, B The expression level of PGM2L1 was higher in breast cancer tissues compared to normal tissues according to Qilu cohort (A, N=73, T=313) and TCGA database (B, N=112, T=1073). **C** The comparison of PGM2L1 level between non-TNBC and TNBC (non-TNBC=1661, TNBC=319). **D** The comparison of PGM2L1 level between non-metastasis and metastasis (M_0=478, M_1=317). **E** Immunohistochemistry of PGM2L1 in TNBC and normal tissues. Scale bars, 500 μm. **F, G** The relationship between PGM2L1 expression and overall survival in breast cancer with Kaplan-Meier survival analysis. Log-rank test.

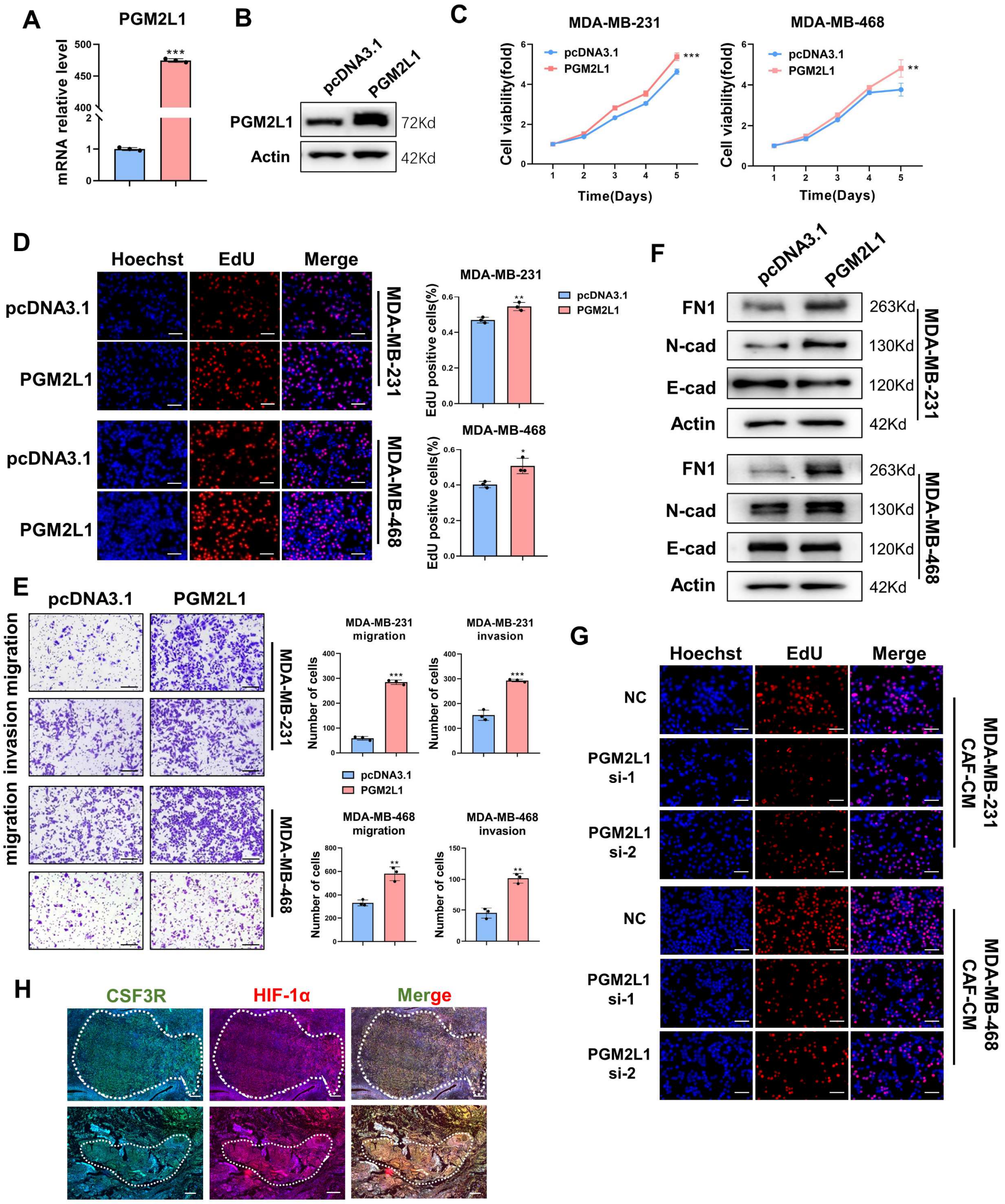

Fig S5. PGM2L1 overexpression promotes the proliferation, migration and invasion of TNBC cells under hypoxia.

A, B Transfection efficiency of PGM2L1-ove plasmid was detected by qRT-PCR (A, n=3) and western blot (B). **C, D** The proliferative ability of TNBC cells overexpressing PGM2L1 was assessed by MTT (C, n=4) and EdU (D, n=3) assays. Scale bars, 100 μm. **E** Transwell assay to assess the migration and invasion abilities of TNBC cells transfecting with PGM2L1-ove plasmid (n=3). Scale bars, 200 μm. **F** The level of EMT-maker proteins was examined by western blot. **G** Representative images of EdU assays

to evaluate the proliferative abilities of TNBC cells treated with hypoxic CAF-CM following PGM2L1 silencing. Scale bars, 100 μm. **H** Fluorescent immunohistochemical staining of the expression of CSF3R and HIF-1α in TNBC tissues. Scale bars, 100 μm. The data are presented as the mean ± SD. * P <0.05, ** P <0.01, *** P < 0.001.

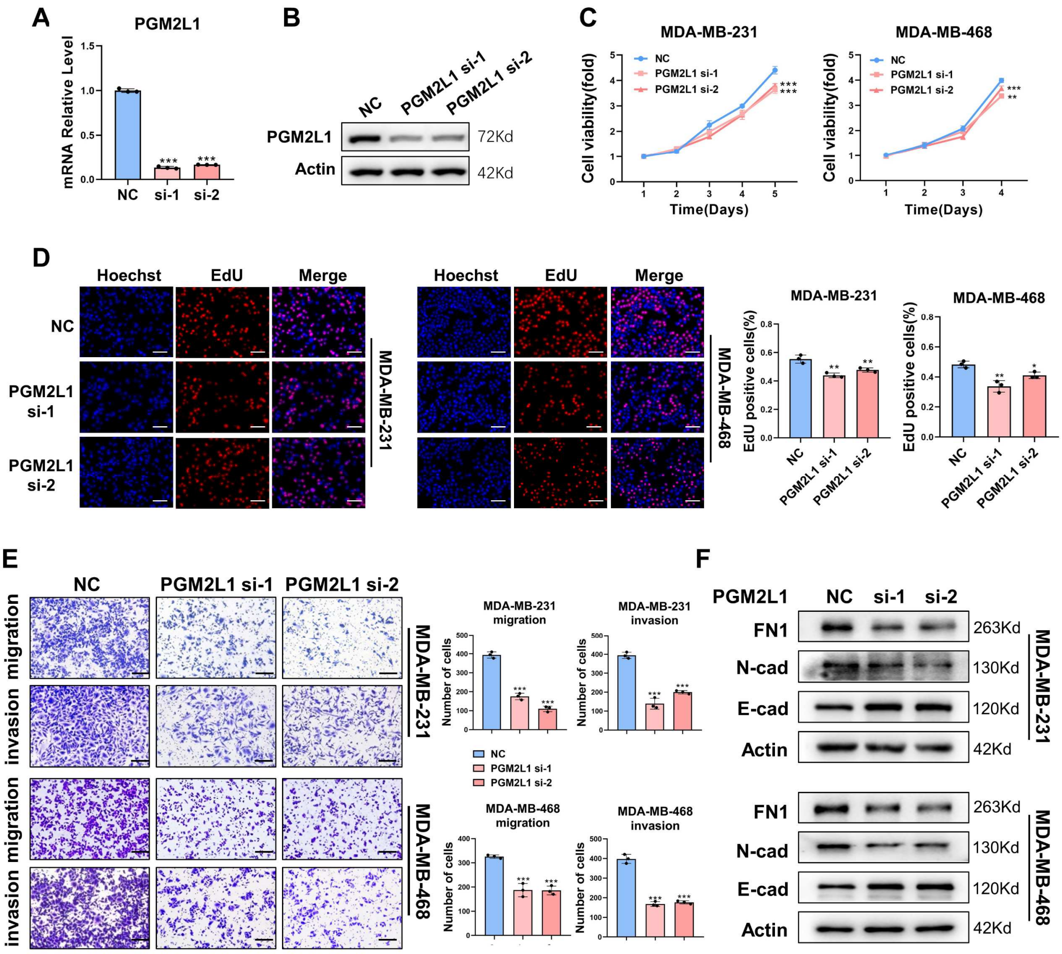

Fig S6. PGM2L1 silencing impairs the proliferation, migration and invasion of TNBC cells under hypoxia.

A, B Transfection efficiency of PGM2L1 siRNA was detected by qRT-PCR (A, n=3) and western blot (B). **C, D** The proliferative ability of TNBC cells transfecting silencing PGM2L1 was assessed by MTT (C, n=4) and EdU (D, n=3) assays. Scale bars, 100 μm. **E** Transwell assay to assess the migration and invasion abilities of TNBC cells transfecting with PGM2L1 siRNA (n=3). Scale bars, 200 μm. **F** The level of EMT-maker proteins was examined by western blot. The data are presented as the mean ± SD. * P <0.05, ** P <0.01, *** P < 0.001.

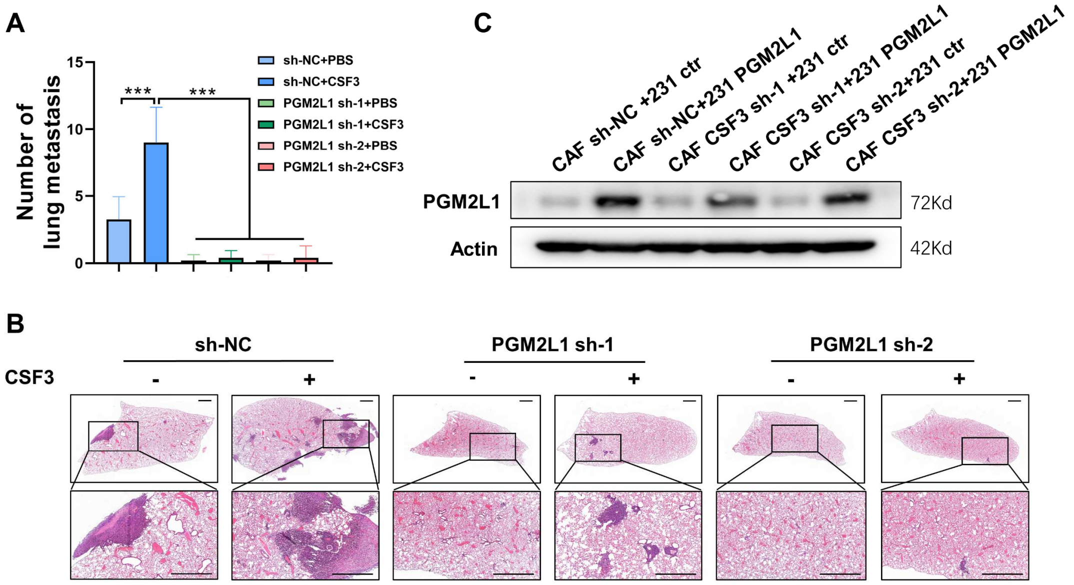

Fig S7. Combined effects of CSF3 and PGM2L1 on tumor growth and lung metastasis in the Balb/c-Nude mice.

A The number of lung metastatic nodules in mice from different groups (n=5 mice). **B** H&E staining images of lung tissues. Scale bars, 1000 μm. **C** The protein level of PGM2L1 in each group was examined by western blot. The data are presented as the mean ± SD. *** $P < 0.001$.

Table 1. siRNA used for transfection

Name	Sense (5’-3’)	Anti-sense(5’-3’)
HIF-1α si	AGUCACCACAGGACAGUAUTT	AUACUGUCCUGUGGUGACUTT
CSF3R si-1	GGACCCAGGAAUCUAUCAUTT	AUGAUAGAUUCCUGGGUCCTT
CSF3R si-2	GACGGAUCCAAGGUUAUGUTT	ACAUAACCUUGGAUCCGUCTT
PGM2L1 si-1	GAACAGAACCAAAGAUAAATT	UUUAUCUUUGGUUCUGUUCTT
PGM2L1 si-2	GGAGAAUGGUUGUUGGAAATT	UUUCCAACAACCAUUCUCCTT
NC	UUCUCCGAACGUGUCACGUTT	ACGUGACACGUUCGGAGAATT

Table 2. Primer sequences used for qRT-PCR

Genes	Forward sequence (5’-3’)	Reverse sequence (5’-3’)
β-actin	CACTGTGCCCATCTACGAG	AATGTCACGCACGATTTCC
CSF3	CGACTTTGCCACCACCATCT	GTAGAACGCGGTACGACACC
HIF-1α	TATGAGCCAGAAGAACTTTTAGGC	CACCTCTTTTGGCAAGCATCCTG
CSF3R	CTTGTGGCCTATAACTCAGCC	CCCACTCAATCACATAGCCCT
PGM2L1	TTACTGCCTCTCACAACCGC	GCGGGCTGGTATCCACTAAA
DPYD	CGGCGGACATCGAGAGTAT	TCCTCGCTCACCAAGAGTCG
GALNT4	TGGCCTATATCTTCGTGGAGC	CTGGAGTTTGCTGGCTTTCC
GLCE	TGAAACAGCAGAAGACAGAGACA	GCCACATTCGCCATAAGCAC
ATP6V1A	TGGAGGGTGACATGGCTACT	GAGGGGTTTACCAGTGCGAA
INPP4B	TGGCTGGCTGCAACGATTT	CTCTTCTCATGCAATCCAGCG
PLD1	AAAATCTGGACACGCGGGAA	GACAGCCGGAGAGATACGTC
IPMK	TCAGCAACAGGTCAGCAAGT	AGCTTCTTCCGTAATGCTGGT
MECOM	TCCGGTACAGCAACATCGTC	TGCCATTCATTCTCTCCTCCAC
GALNT5	TTGGAACATACGACCCTGGC	CCACCACACATCCACACCTT

PFKL	GGGAGGTGAGAACATCAAGCA	CCAATGATAGTGCCGCCCAG
ENO1	AACCCAAAGAGGATCGCCAA	CCCGAACGATGAGACACCAT
PKM1F	CAGCACCTGATAGCTCGTGA	TGAGGCTCGCACAAGTTCT
PDK1	GCCACTATGGAACACCATGC	CCTCATTACCCAGCGTGACA
HK2	GCCCGCCAGAAGACATTAGA	GCTCAGACCTCGCTCCATTT
PKM2	AATCACGCTGGATAACGCCT	TCGGCACCTTTCTGCTTCAC

Table 3. Antibodies used for western blot and IHC

Name	Supplier	Catalog#	Application
α-SMA	Proteintech [a]	55135-1-AP	WB（1:500）IHC（1:300）
FAP	Affinity [b]	AF5344	WB（1:500）
FSP1	Abclonal [c]	A1631	WB（1:500）
FN1	Proteintech	15613-1-AP	WB（1:500）
Vimentin	Servicebio [d]	GB11192	WB（1:500）
CSF3R	Proteintech	18310-1-AP	WB（1:200）
p-ERK	Affinity	AF1015	WB（1:1000）
ERK	Affinity	AF0155	WB（1:1000）
p-AKT	Affinity	AF0016	WB（1:500）
AKT	PTM BIO [e]	PTM-5191	WB（1:500）
p-STAT3	Affinity	AF3293	WB（1:500）
STAT3	Affinity	AF6294	WB（1:500）
N-cadherin	Proteintech	22018-1-AP	WB（1:500）
E-cadherin	Proteintech	20874-1-AP	WB（1:500）
PGM2L1	Proteintech	13942-1-AP	WB（1:500）IHC（1:100）
Ki-67	Proteintech	28074-1-AP	IHC（1:500）
Actin	Affinity	T0022	WB（1:1000）

[a] Proteintech, Chicago, IL, USA

[b] Affinity, Changzhou, China

[c] Abclonal, Wuhan, China

[d] Servicebio, Wuhan, China

[e] PTM BIO, Hangzhou, China

Table 4. Reagents used in the experiment

Name	Supplier	Catalog	Application
CSF3	R&D System [a]	214-CS	50ng/ml
2-DG	Med Chem Express [b]	HY-13966	500μM
ERK inhibitor	Med Chem Express	SCH772984	2.5μM
AKT inhibitor	Med Chem Express	MK-2206	5μM
Stat3 inhibitor	Med Chem Express	NSC 74859	5μM

[a] R&D System, Minneapolis, MN, USA

[b] Med Chem Express, Rahway, NJ, USA
