# Supplementary figures and images for: Cancer-associated fibroblasts secrete CSF3 to promote TNBC progression via enhancing PGM2L1-dependent glycolysis reprogramming
Source: Cell Death Dis. 2025 Apr 4;16(1):249. doi: 10.1038/s41419-025-07580-6 (PMC11971334; doi:10.1038/s41419-025-07580-6)

**Fig3-D**

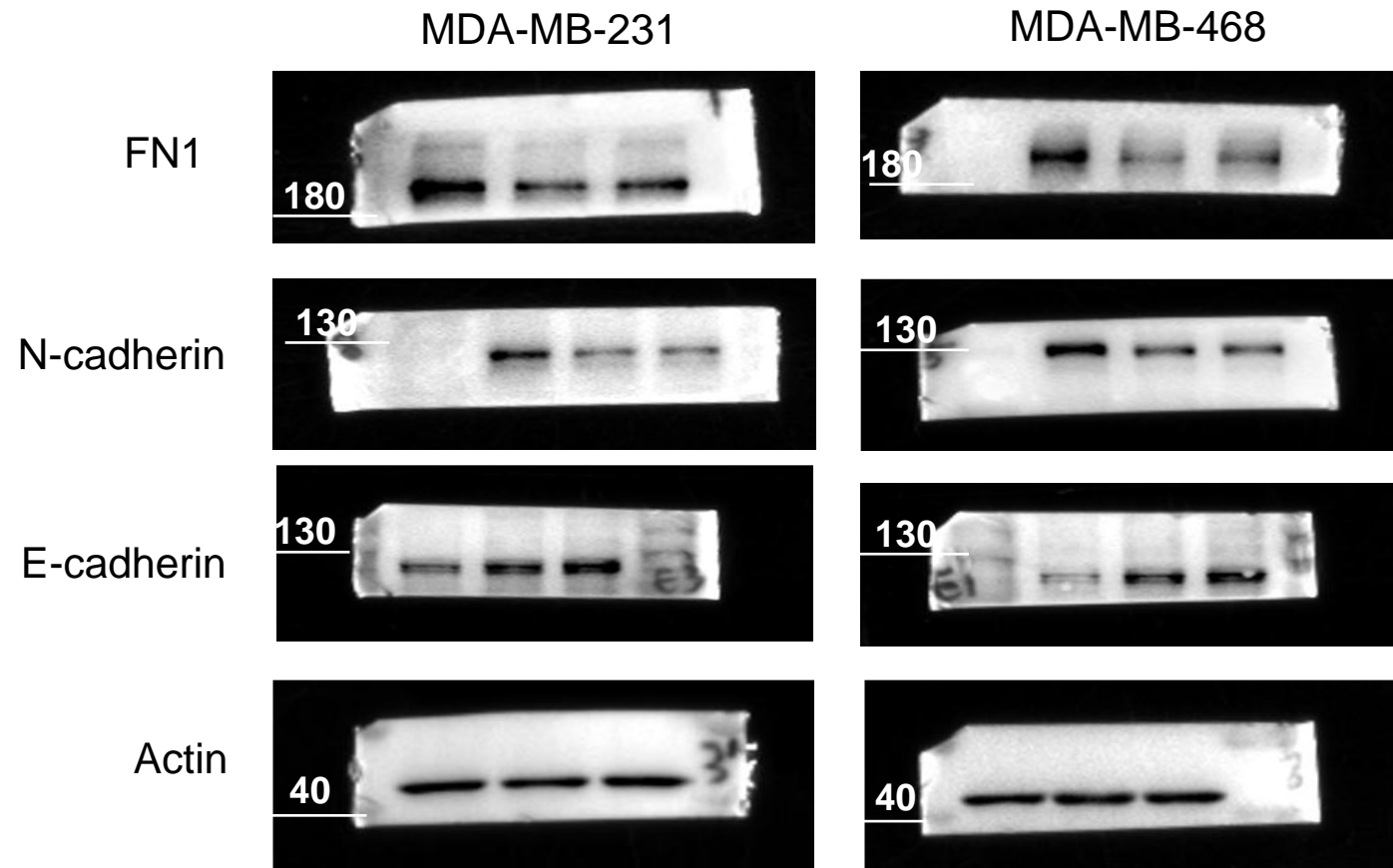

**Fig4-C**

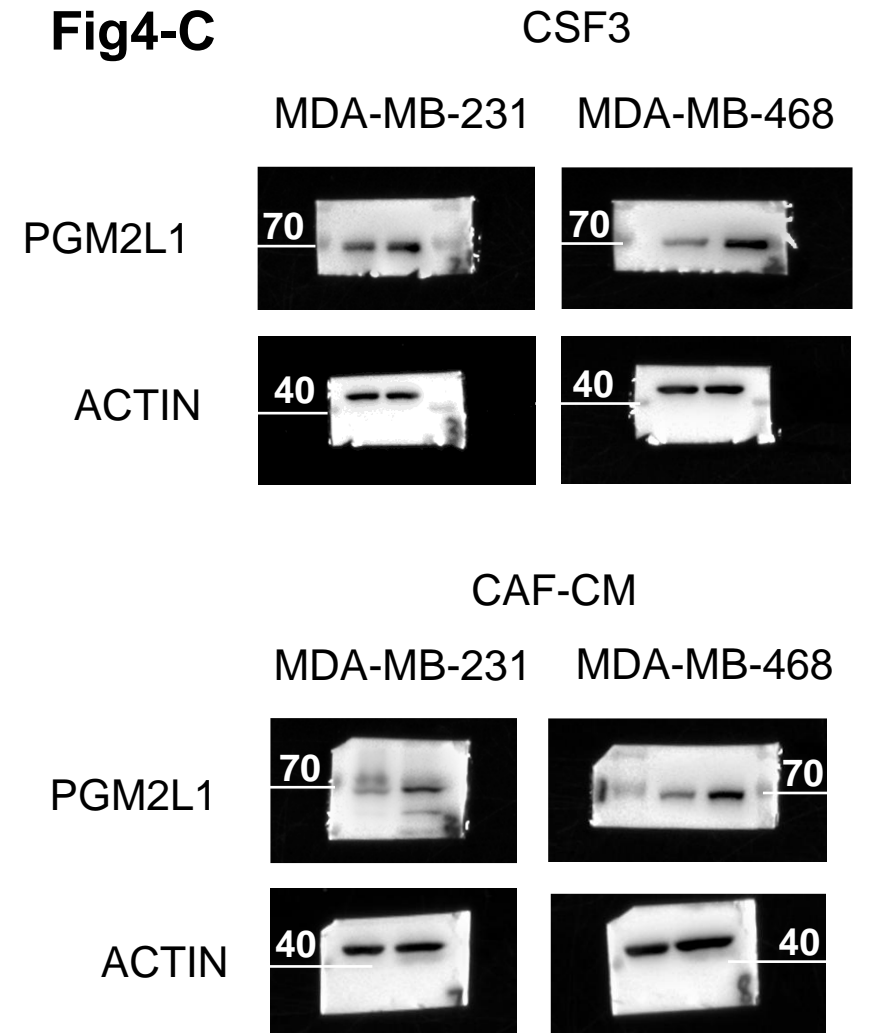

**Fig4-E**

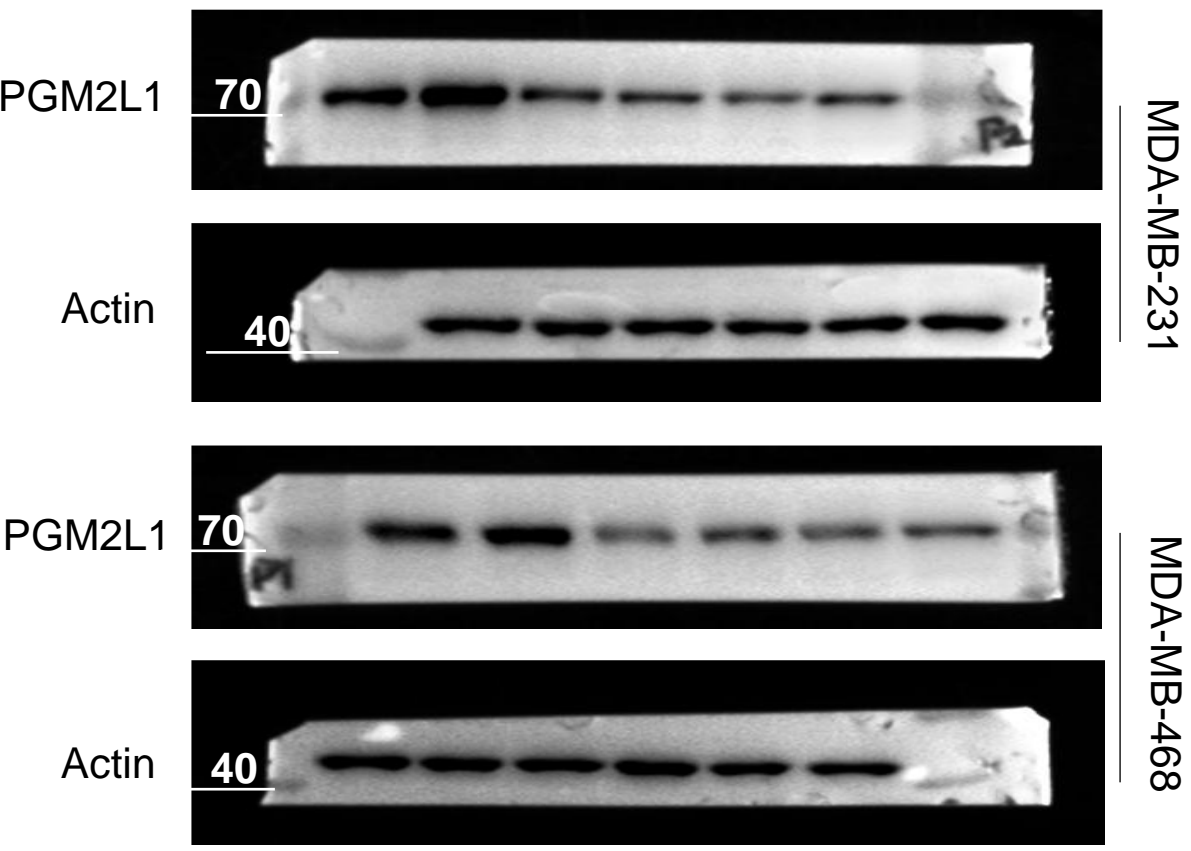

**Fig5-I**

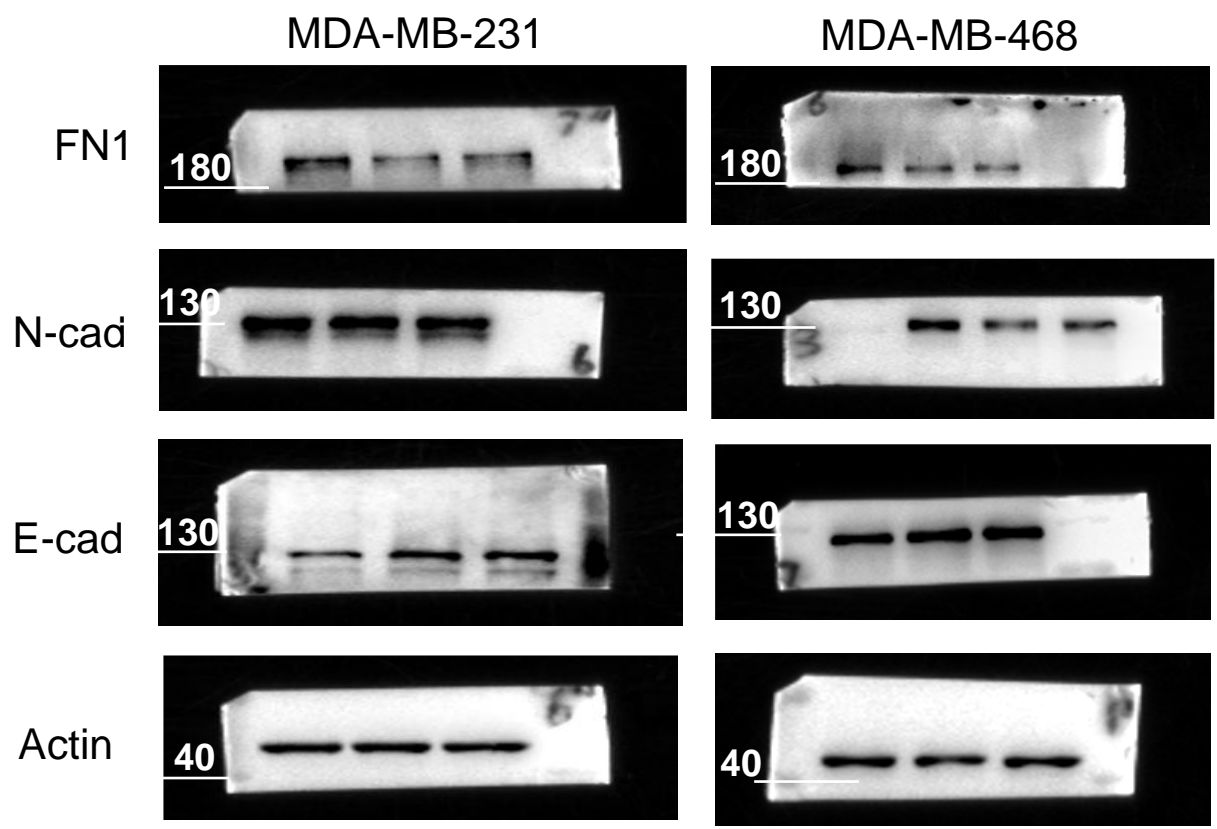

**Fig7-E**

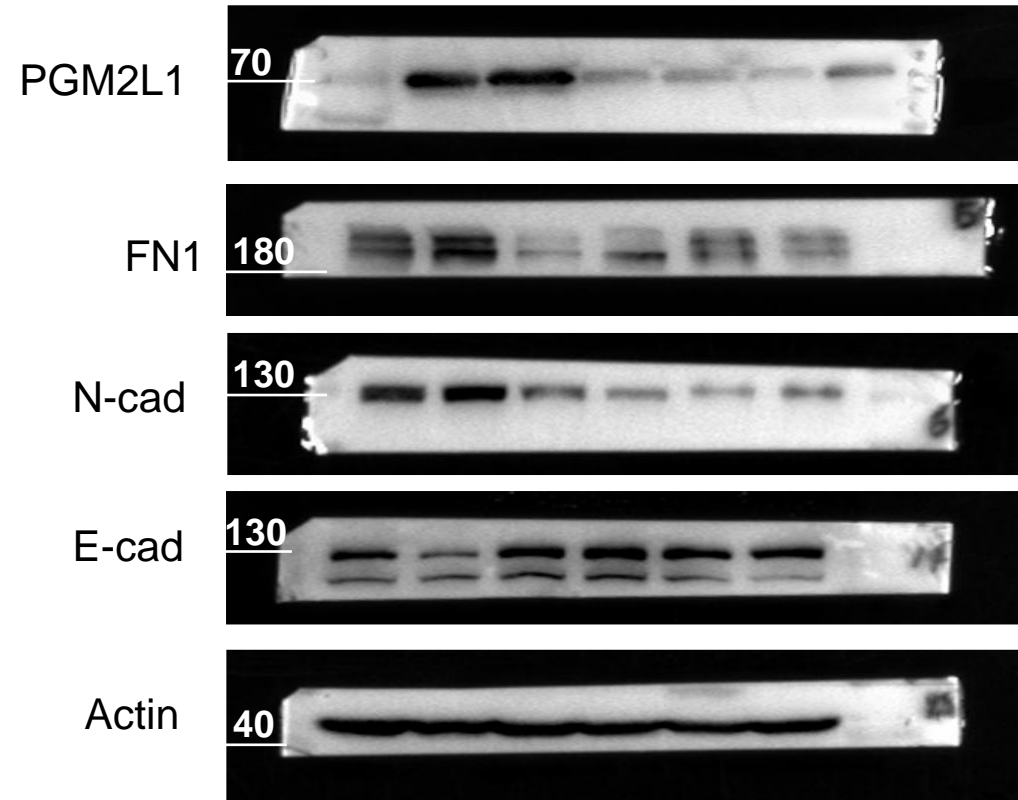

**Fig S1-B**

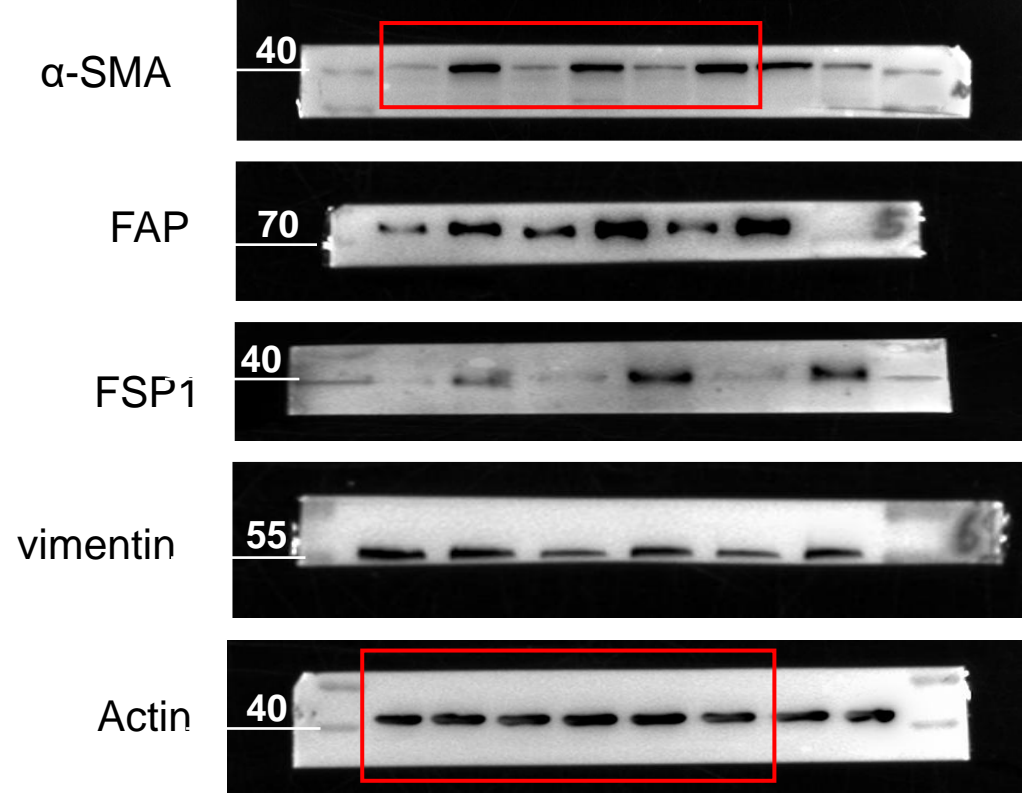

**Fig S2-B**

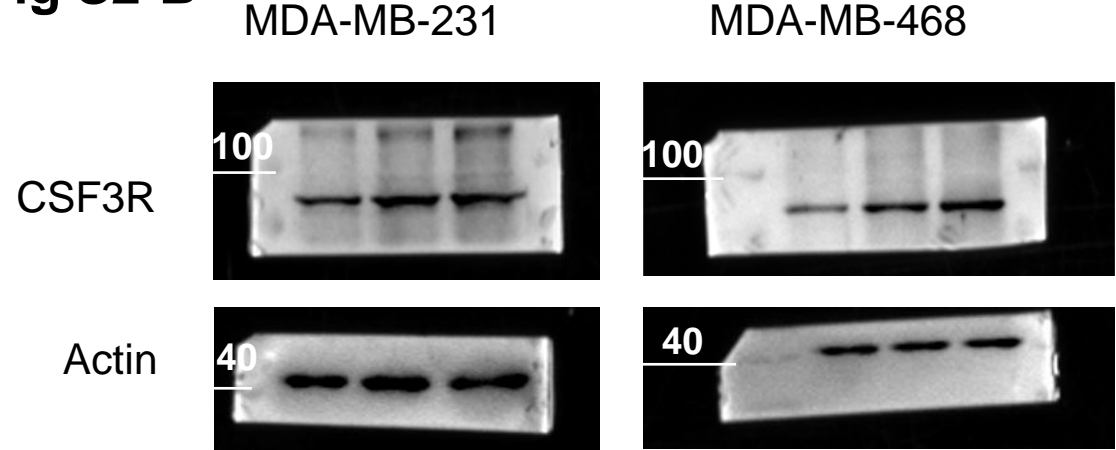

**Fig S2-D**

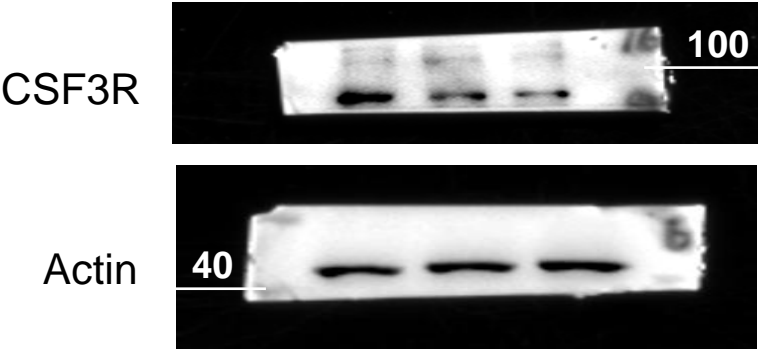

**Fig S2-E**

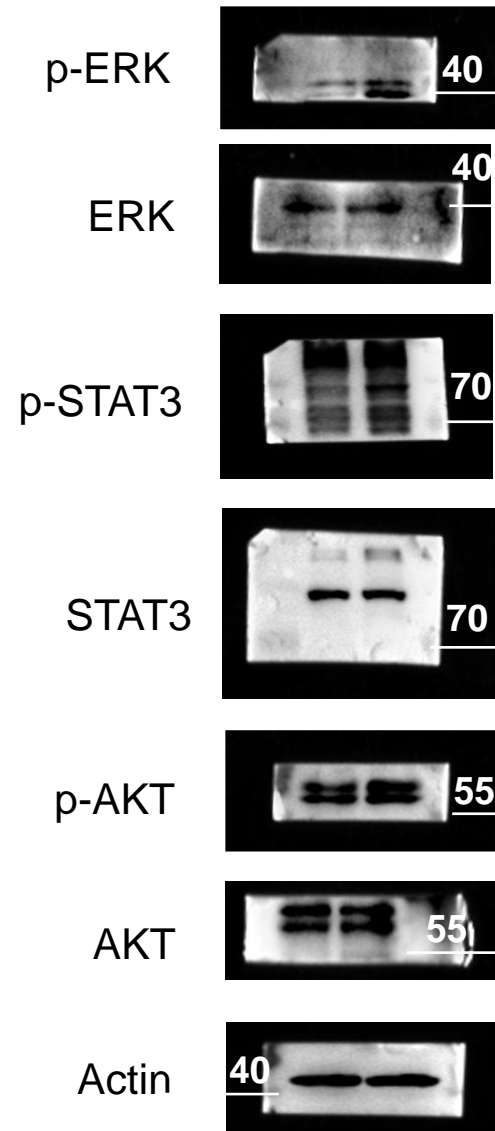

**Fig S3-E**

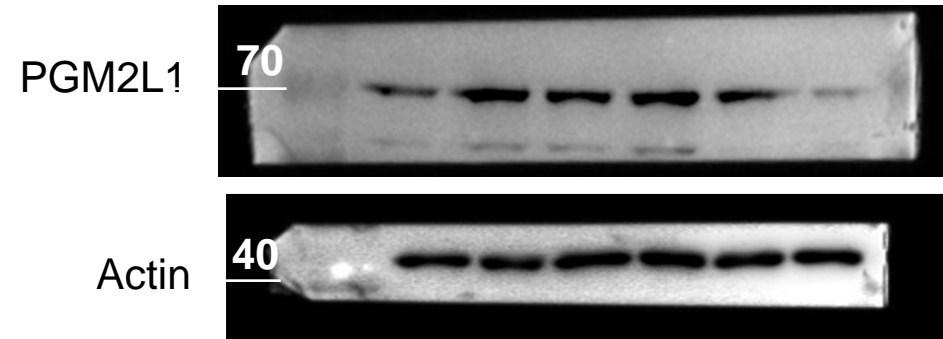

**Fig S5-B**

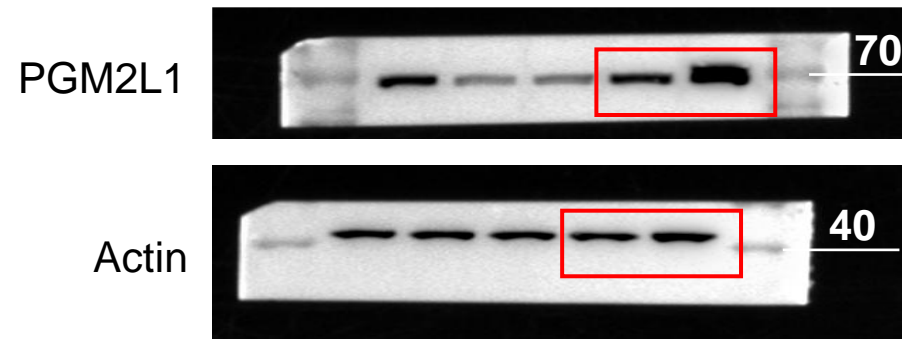

**Fig S5-F**

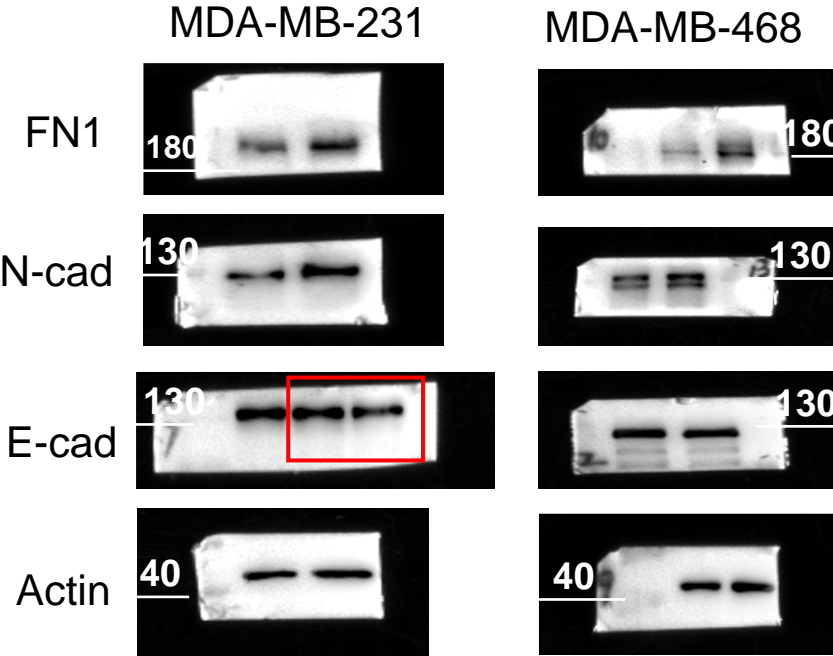

**Fig S6-F**

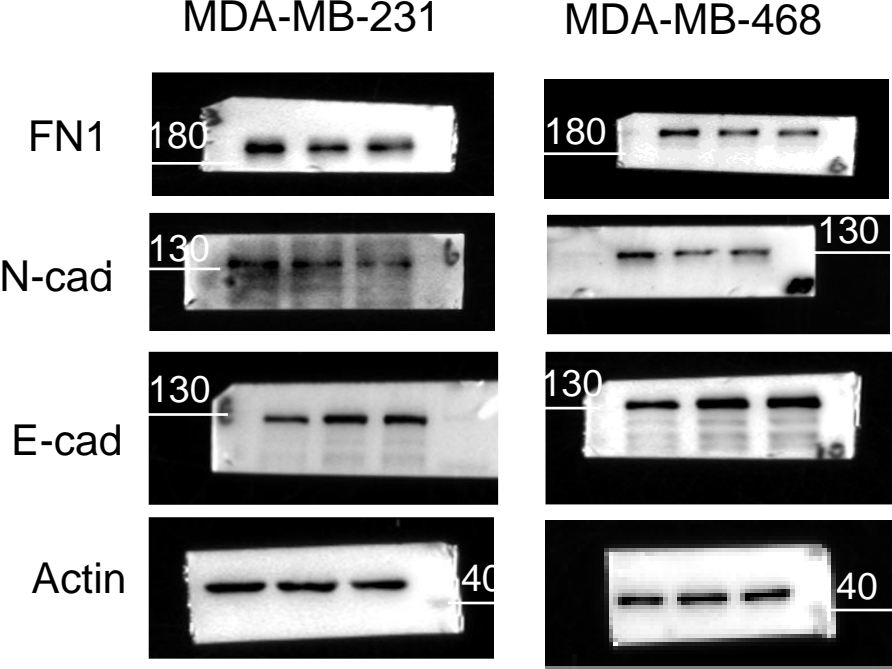

**Fig S6-B**

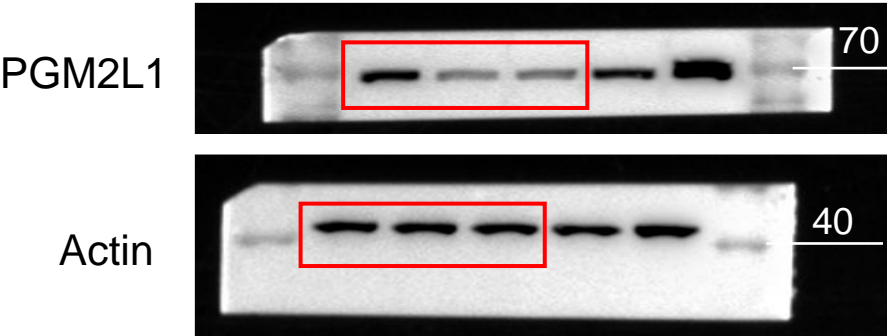

**Fig S7-C**

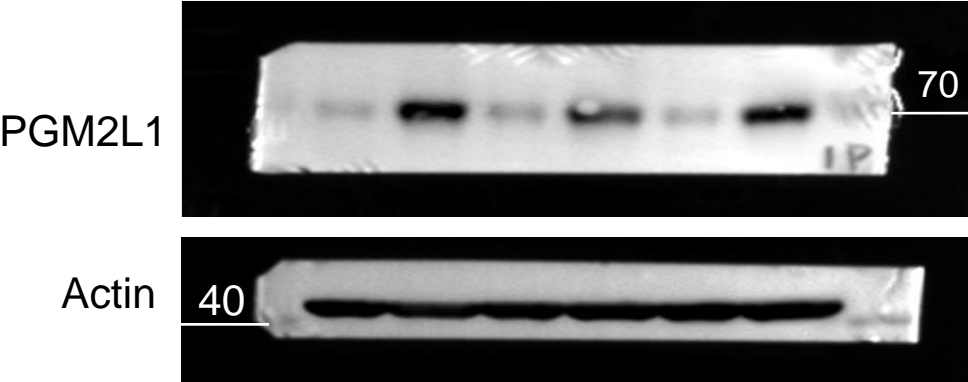

Supplement: Supplementary file 2 — Western blots [file 41419_2025_7580_MOESM2_ESM.pdf]
